# Endometrial cancer diagnostic and prognostic algorithms based on proteomics, metabolomics, and clinical data: a systematic review

**DOI:** 10.3389/fonc.2023.1120178

**Published:** 2023-04-06

**Authors:** Andrea Romano, Tea Lanišnik Rižner, Henrica Maria Johanna Werner, Andrzej Semczuk, Camille Lowy, Christoph Schröder, Anne Griesbeck, Jerzy Adamski, Dmytro Fishman, Janina Tokarz

**Affiliations:** ^1^ Department of Gynaecology, Maastricht University Medical Centre (MUMC), Maastricht, Netherlands; ^2^ GROW – School for Oncology and Reproduction, Maastricht University, Maastricht, Netherlands; ^3^ Institute of Biochemistry and Molecular Genetics, Faculty of Medicine, University of Ljubljana, Ljubljana, Slovenia; ^4^ Department of Gynaecology, Lublin Medical University, Lublin, Poland; ^5^ Sciomics GmbH, Heidelberg, Germany; ^6^ Department of Biochemistry, Yong Loo Lin School of Medicine, National University of Singapore, Singapore, Singapore; ^7^ Institute of Experimental Genetics, Helmholtz Zentrum München, German Research Center for Environmental Health, Neuherberg, Germany; ^8^ Institute of Computer Science, University of Tartu, Tartu, Estonia; ^9^ Quretec Ltd., Tartu, Estonia; ^10^ Institute for Diabetes and Cancer, Helmholtz Zentrum München, German Research Center for Environmental Health, Neuherberg, Germany; ^11^ German Center for Diabetes Research (DZD), Neuherberg, Germany

**Keywords:** endometrial cancer, proteomics, metabolomics, biomarker, machine learning

## Abstract

Endometrial cancer is the most common gynaecological malignancy in developed countries. Over 382,000 new cases were diagnosed worldwide in 2018, and its incidence and mortality are constantly rising due to longer life expectancy and life style factors including obesity. Two major improvements are needed in the management of patients with endometrial cancer, i.e., the development of non/minimally invasive tools for diagnostics and prognostics, which are currently missing. Diagnostic tools are needed to manage the increasing number of women at risk of developing the disease. Prognostic tools are necessary to stratify patients according to their risk of recurrence pre-preoperatively, to advise and plan the most appropriate treatment and avoid over/under-treatment. Biomarkers derived from proteomics and metabolomics, especially when derived from non/minimally-invasively collected body fluids, can serve to develop such prognostic and diagnostic tools, and the purpose of the present review is to explore the current research in this topic. We first provide a brief description of the technologies, the computational pipelines for data analyses and then we provide a systematic review of all published studies using proteomics and/or metabolomics for diagnostic and prognostic biomarker discovery in endometrial cancer. Finally, conclusions and recommendations for future studies are also given.

## 1 Introduction

### 1.1 Endometrial cancer – The need for minimally invasive diagnostic and prognostic biomarkers

Endometrial cancer (EC) is the most common gynaecological neoplasm in developed countries, and over 382,000 new cases were diagnosed worldwide in 2018 (1). In general, EC is diagnosed in postmenopausal women (85% of cases) and its incidence is rising due to longer life expectancy and life style associated risk factors. Women with BMI above 35 have an odds ratio of 5.7 for developing EC, with an increase of 1.1 odds ratio per BMI unit (2, 3). Exposure to unopposed estrogens or tamoxifen or genetic aberrations associated with Lynch syndrome confer a cumulative risk up to 70% (4, 5). Finally, endocrine disruptors and other environmental pollutants can also increase EC risk (6, 7). Therefore, an alarmingly high number of women in the general population is exposed to risk factors for developing EC.

In this context, screening programs would be extremely beneficial for these women, but, unfortunately, no minimally- or non-invasive diagnostic tool for EC exists today, and diagnosis relies on invasive endometrial biopsy and pathology investigation.

A second unmet clinical need in EC is the necessity to accurately stratify patients. EC is diagnosed at an early FIGO stage in 80% of the cases, and the five-year survival of FIGO stage 1a is around 95%. However, a proportion of women diagnosed with early-stage EC develop recurrent disease, which dramatically decreases survival rates (8). This represents a challenge as recent projections indicate that the worldwide EC mortality will increase by 70% by 2040 (Global Cancer Observatory, World Health Organisation - https://gco.iarc.fr).

Therefore, prognostic biomarkers to reliably predict patient prognosis are needed, both prior to any intervention - to decide on the most appropriate treatment and if needed optimally plan the surgical procedure - as well as post-operatively, to define the most appropriate adjuvant treatment, and avoid over-treatment and under-treatment. A number of prognostic markers like histological assessment of tumour type and grade, hormone receptor status, PTEN expression, mismatch repair proteins (MLH1, PMS2, MSH2, MSH6), *POLE* exon 3 mutation, *CTTNB1* mutation, L1CAM overexpression, and *TP53* aberrations allow stratification of patients according to their risk of recurrence (9–20). In particular, the recent introduction of The Cancer Genome Atlas (TCGA) molecular classification improved the risk stratification at the postsurgical (21, 22), but also improved the concordance between presurgical biopsy and pathology assessment at hysterectomy (23), which has been a problem in the past (24). This classification groups EC patients in four clusters with distinct prognosis and a number of studies demonstrated the reliability and the clinical applicability of this classification using surrogate analyses (i.e., IHC and *POLE* gene mutation analyses). Patients with *POLE* mutated tumours have the best prognosis, followed by mismatch repair deficient tumours and with the final groups having an intermediate and the worse prognosis (no specific molecular profile and p53 mutated, respectively) (14). Recently, also classification methods fully based on IHC, hence applicable also in centres with limited access to molecular infrastructures, showed robustness and reliability (19).

Nevertheless, these methods require invasive biopsies, and women consider the presurgical biopsy procedures discomforting and painful (25). Therefore, non- or minimally-invasive prognostic tools applicable presurgically are urgently needed.

Proteomic and metabolomic profiles are attractive approaches for identifying biomarkers that can be detected in tissues or body fluids obtained *via* non-, minimaly or semi--invasive procedures. The purpose of the present review is to explore the current research on the use of proteomics and metabolomics in the context of EC. This review provides a brief introduction to the wet-lab technologies, the computational pipelines for data analyses and a systematic review of all published studies aimed at using proteomics and/or metabolomics for diagnostic and prognostic biomarker discovery in EC. This is followed by conclusions and recommendations for future studies.

### 1.2 Proteomic and metabolomic approaches for biomarker discovery

Proteomics and metabolomics represent fields that have grown significantly in the last decades, thanks to the important technological advances that allow accurate and sensitive analyses. Both approaches have been extensively used for biomarker discovery in various disorders (26–31).

#### 1.2.1 Targeted and non-targeted proteomics

Large-scale proteomics mainly relies on two different methodological approaches, namely immune-based, targeted protein microarrays and (non-targeted and targeted) mass spectrometry (MS). Making use of antibody-protein specific binding, protein microarrays can be seen as miniaturized conventional assays, thereby allowing multiplexing and high throughput. Protein microarrays relevant for biomarker discovery fall into three categories: analytical microarrays, reverse phase protein array (RPPA), and bead-based microarrays. Analytical protein microarrays are also called capture or antibody microarray because proteins from complex protein lysates are captured by antibodies or aptamers, which have been previously immobilized on the surface of an array. Conversely, RPPA is based on the immobilization of complex samples on a surface and subsequent probing by pre-selected antibodies. Bead-based microarrays use capture antibodies immobilised on microbeads combined with secondary, detection antibodies. Protein microarrays are highly sensitive and highly specific assays, which allow relative quantification among different clinical sample groups. While multiplexity is usually higher in analytical microarrays compared to RPPA and bead-based microarrays, all methods share simple sample processing allowing high throughput. A further immune-based method, Olink technology, uses antibodies that are labelled with ssDNA and detect proteins in a sample by proximity extension assay (PEA). Pairwise antibodies are linked with complementary ssDNA which upon binding the target protein are hybridized and extended using a DNA polymerase. Despite being targeted hypothesis-driven approaches, antibody-based technologies like protein arrays are solid and promising tools for biomarker discovery and verification (32–34).

Mass spectrometry measures mass-to-charge ratios of ionized peptides in order to analyse proteins. Ionization of proteins can be achieved by electrospray ionization (ESI) or by matrix-assisted laser desorption/ionization (MALDI). ESI allows the creation of ions in solution, while in MALDI, ions are created by laser light pulsing on matrix embedded proteins. A variation of MALDI is SELDI (surface-enhanced laser desorption/ionization), where the proteins are applied on a modified matrix surface allowing binding of specific proteins or proteins classes (35). Mass analysis of proteins is primarily conducted using TOF (time-of-flight) or quadrupoles. Sample preparation for mass spectrometry is a complex process. Upon cellular lysis, it includes subcellular fractionation, depletion of highly abundant proteins, enrichment of target proteins, denaturation and protein digestion. Resolving and denaturation of proteins can also be achieved by SDS polyacrylamide gel electrophoresis, 1D or 2D polyacrylamide gel electrophoresis (PAGE) or difference gel electrophoresis (DIGE). Mass spectrometry is not inherently quantitative but different types of labelling (isobaric tags for relative and absolute quantitation - iTRAQ; isotope-coded affinity tag - ICAT, stable isotope labelling by amino acids in cell culture - SILAC) allow relative and absolute quantification. Label-free quantification, based on signal intensity, is an alternative, cost-efficient option but with a relatively low throughput (36).

Non-targeted mass spectrometry is widely used for biomarker discovery because of its suitability for hypothesis-free approaches. Due to the complexity of the workflow, the number of samples analysed in a discovery setting is usually quite limited, especially when plasma samples are used. Furthermore, fractionation, depletion of high abundant proteins or digestion could bias the results and limit the sensitivity in the untargeted approach. In general, only a small number of candidates undergo clinical validation using orthogonal platforms and even fewer are tested in clinical studies (37, 38) as these studies need first the transition of MS data into immunobased assays to analyse a sufficiently large and statistically relevant number of samples. In this regard, protein microarrays for discovery present the advantage that such translation is not necessary (33).

Proteomics displays a large panel of different tools, which can be combined for discovery and validation phases and subsequently integrated in multi-omics approaches (39).

#### 1.2.2 Targeted and non-targeted metabolomics

Metabolomics is the most recent ‘omics’-technology and strives to measure ideally all metabolites in a given biological sample (40). Since metabolites are final downstream products of all cellular processes, metabolomics is closest to the phenotype compared to the other ‘omics’-techniques.

Similar to proteomics, two approaches with different objectives are used (41), namely non-targeted and targeted metabolomics. Non-targeted metabolomics (profiling metabolomics) is a hypothesis-free approach, which aims to detect simultaneously as many metabolites as possible. Depending on the analytical platform, non-targeted metabolomics reveals metabolites from a wide range of metabolite classes (42), which are annotated after the measurement. Thus, the detection of unknown metabolites not yet annotated in metabolite databases is common in non-targeted metabolomics. Although being comprehensive, non-targeted metabolomics does not allow absolute quantification, but can provide at best only semiquantitative results (42).

Targeted metabolomics is hypothesis-driven and aims to quantify the absolute concentrations of a predefined set of metabolites (42). Since all measured metabolites are pre-selected, a standard calibration curve for accurate quantification can be prepared for each metabolite. Stable-isotope labelled internal standards are added at known concentrations to all samples, allowing compensation for any analytical interferences. With its advantages such as validated analytical performance and the results delivered in absolute concentrations, targeted metabolomics is often used for biomarker validation (42). However, the limited number of simultaneously quantified metabolites in targeted metabolomics increases the risk of missing relevant biological processes.

Metabolomic approaches usually use MS or nuclear magnetic resonance spectroscopy (NMR). While MS offers high mass accuracy, high resolution, high dynamic range and high sensitivity (43), NMR is less sensitive but is superior in terms of structural information content, robustness, and reproducibility (44, 45). However, current analytical methods are not able to cover the entire metabolome (46). To achieve a high metabolite coverage combined with quantitative data, the integration of different metabolomic techniques (multiplatform approaches) is necessary (46, 47).

### 1.3 Bioinformatics and statistical approaches for constructing diagnostic and prognostic algorithms

**Figure 1:**
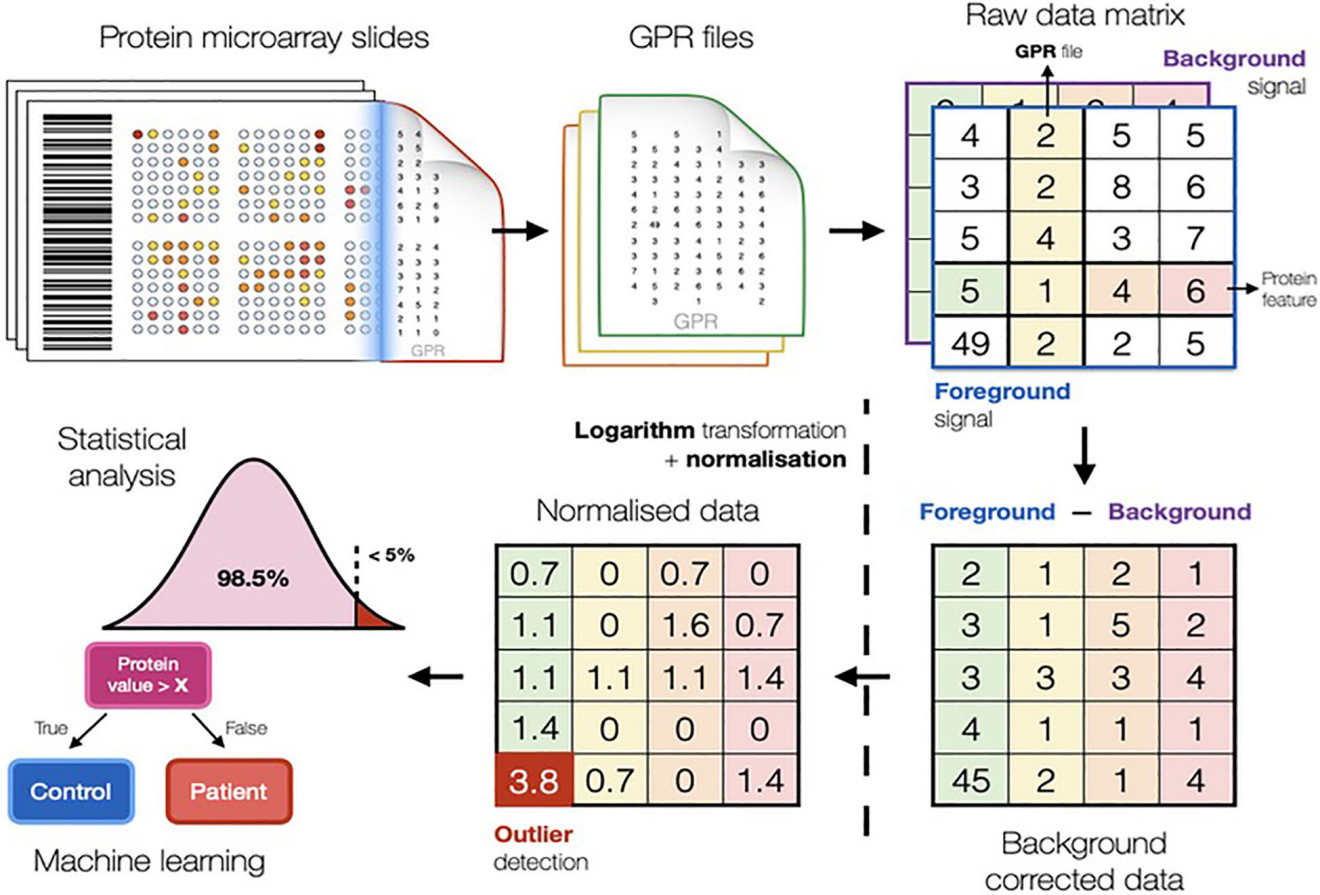
Scheme illustrating the typical computational methods used in biomarker discovery studies to analyse data. The pipeline in figure refers to the conventional protein microarray analysis (Fishman et al. https://arxiv.org/abs/2201.06074).

Data from proteomic and metabolomic experiments can reveal molecules that can possibly serve as diagnostic or prognostic markers. However, even if well-designed and executed, experiments often result in noisy, biased and incomplete data due to a multitude of uncontrollable factors. Therefore, thorough data analysis needs to be performed to eliminate technical noise, while preserving genuine biological variation between samples. A set of computational methods used to analyse data are typically bundled together into one unified data analysis pipeline (**Figure 1**), which treats raw data files as an input while providing the list of potential biomarkers as the output.

Proteomic and metabolomic raw data is first processed by background correction, signal transformation, outlier detection, and normalisation. Pre-processing is essential to minimise unwanted technical bias and enable comparisons of samples. Further, integration of clinical information enables comparisons of average metabolite and protein signals between phenotypic groups of interest (48–50).

Background correction addresses different effects in proteomics and metabolomics. In protein microarrays, it is challenging to correctly quantify the fluorescent signal produced by the biological reaction avoiding local residuary background (51–53). In metabolomics, background (or baseline) correction is used to eliminate low frequency artefacts and differences generated by the measuring instrument (54). Data log-transformation is common practice, as this renders fold changes symmetric around zero, reduces potential skew in the data and provides a good approximation for the normal distribution, which is a prerequisite for most computational methods (52, 55), especially for linear models. Following background correction, outlier detection is performed by the three standard deviations technique and subsequently removed or replaced.

In large-scale studies based on MS proteomics or metabolomics, samples will be distributed into several analytical batches, which may introduce instrumental variabilities into the data set. Such batch effects can be very destructive as they render comparison between phenotypic groups ineffective. Normalisation strategies for metabolomic and proteomic experiments make use of control samples and control molecules (52, 56, 57), which are usually assumed to exhibit constant signal levels. Any differences in signal values are considered to be technogenic and thus, corrected for. The most popular normalisation strategies are global scaling, quantile normalisation, cyclic loess and the ones involving linear models (56).

After appropriate pre-processing, the data is used for statistical analysis, where a large number of techniques are available. Characteristics of data and the research question determine the choice of the statistical method. In biomarker discovery studies, molecules that can reliably distinguish between two (or more) groups, like disease versus controls, are referred to as significantly differential and can be used as biomarker candidates. The process of identifying such molecules is termed differential analysis (52, 58). Classical univariate statistics such as Student’s t-test (requiring normal data distribution) or Mann-Whitney U test (non-parametric test that does not rely on parameterized data distribution) can be used for differential analyses. Differential analyses have a low probability (usually less than 5%) to deliver significant results by mistake; however, if repeated multiple times, as for omics studies, can result in the generation of false significant hits. Therefore, the number of tests performed needs to be taken into account. The simplest and one of the most popular methods for multiple testing correction is ‘Bonferroni correction’ (59), which adjusts the p-value threshold by dividing it by the number of tests. This is a conservative approach that may result in a high number of potential biomarkers being ignored, hence, less stringent methods can be considered (like Benjamini and Hochberg False Discovery Rate correction) that keeps the number of falsely significant results at a predefined level (e.g., 5%).

While classical statistical methods analyse the significance of each molecule of interest independently (60), machine learning algorithms are able to efficiently assess the predictive performance of multiple proteins, metabolites, features and even their combinations. Machine learning is a field of computer science that studies algorithms capable of learning valuable relationships from data without being explicitly programmed. Myriads of machine learning algorithms have been developed over the past years (61) and are frequently used in biology to discover biomarkers for various diseases (49, 62). The most popular machine learning methods are decision tree (63), support vector machine, random forest (64) and gradient boosting machines (65).

It can be challenging to build reliable machine learning models, because most model algorithms can learn random patterns that can only explain data these models were exposed to. This phenomenon is known as overfitting and might cause models to report completely irrelevant biomarkers and thus, render the entire study obsolete. In order to account for potential overfitting and keep its influence at minimum, various strategies have been proposed (66). One of the most important techniques is k-fold cross validation. By using only one part of the data to build a model (training set) and the remaining part to assess its performance (test set), researchers can be confident that the biomarkers identified by the model are not random fluctuations in the training data.

## 2 Methods

### 2.1 Study design

With this systematic review we aimed to respond to the following question: Can proteomics and metabolomics contribute to identification of biomarkers for diagnostics and prognostics in EC? The review was conducted according to the PRISMA guidelines (67) and is registered at the ‘International prospective register of systematic reviews’ (PROSPERO, Registration number CRD42022245880).

### 2.2 Search strategy, data extraction and quality assessment

We performed a systematic search of the literature in the PubMed^®^ and OVID^®^ Embase databases on July 20, 2022 using the search terms listed in **Supplementary Table S1**. We focused on proteomic and metabolomic studies performed in physiological fluids and tissue samples. There was no restriction on publication date. Reports were retrieved, and titles/abstracts were screened according to the inclusion and exclusion criteria (**Table 1**) independently by authors AR and TLR. Disagreements were discussed and consensus was reached. The search strategy is provided in **Table 2**.

**Table 1:** Inclusion and exclusion criteria.

**Inclusion criteria**	research papers, papers in English, studies in humans, blood (plasma or serum), urine, other physiological fluids, tissue samples, at least 10 subjects per study group.
**Exclusion criteria**	abstracts, review papers, papers in other languages, studies in animals, studies in cell lines, studies including only unidentified metabolites, epidemiological studies, studies evaluating drug effects.

**Table 2:** Search strategy for identification of manuscripts in Pubmed and OVID Embase.

Search Query	Search results	Selected manuscripts	Additional manuscripts	Included
Endometrial cancer and proteomics*	**746 total** 570 PubMed 176 OVID **275 duplicates** **471 total** **224 removed/titles** **247 total** 175 removed/abstract **Total 72**	**23** 49 removed after full-text reading	**0**	**23**
Endometrial cancer and metabolomics*	**214 total** 89 PubMed 125 OVID **43 duplicates** **171 total** **70 removed/titles** **101 total** 70 removed/abstract **Total 31**	**31** 4 removed after full-text reading	**2**	**29**

*Search strategy is provided as **Supplementary Table S1**.

The selected reports were read in detail and the following relevant data was extracted (when applicable): author and year of publication, country, fundings; sample: tissue (kind), plasma, serum or other body-fluid; study design; methods: omics approach, targeted/nontargeted proteomics/metabolomics; analytical methods; patient selection: case/control, stratification of patients according to reference test; patient characteristics: number of patients, characteristics of the enrolled patients with EC (e.g., mean age, body mass index [BMI], type of EC, histological differentiation, FIGO stage, menopausal status) and control patients or healthy women (e.g., mean age, BMI, diagnosis, menopausal status); study phase and statistical methods: discovery, validation phase, machine learning approaches used; differentially abundant proteins and metabolites in the study groups; diagnostic characteristics (e.g., sensitivity, specificity, area under the curve [AUC], positive predictive value [PPV], negative predictive value [NPV]) or prognostic characteristics (overall survival [OS], disease-free survival [DFS]), and hazard ratios [HR]; diagnostic or prognostic models; disclosures: affiliations with industry, industrial funds, patents.

Reporting was performed under the guidance of the PRISMA diagnostic test accuracy checklist (67). The risk of bias and quality of individual diagnostic accuracy studies were assessed following the QUADOMICS tool, an adaptation of QUADAS (68) that was designed specifically for omics studies (69) (**Supplementary Table S2**). This tool focuses on study design, patient selection, index test, reference standards, flow of timing, pre-analytical and analytical procedures, and statistical analysis and nine questions per study were specifically answered (**Supplementary Tables S2–S4**). Additional potential financial, commercial and conflict of interest biases were further examined (**Supplementary Tables S5**).

## 3 Results

**Figure 2:**
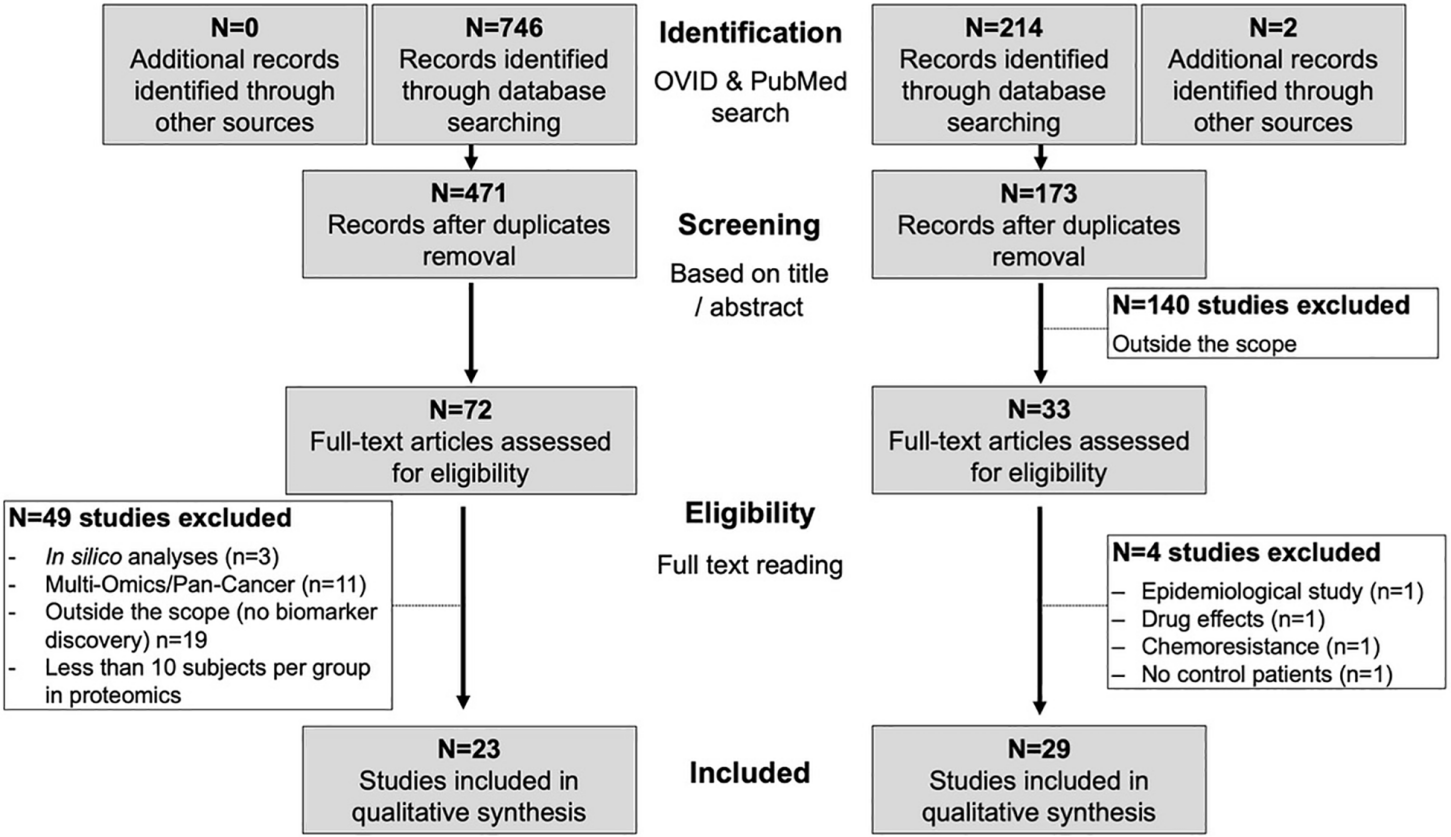
Workflow of the systematic search and paper selection.

Systematic literature search led to the identification of 52 studies in EC, 23 on proteomics and 29 on metabolomics (**Figure 2**).

### 3.1 Evaluation of the quality of published studies

**Figure 3:**
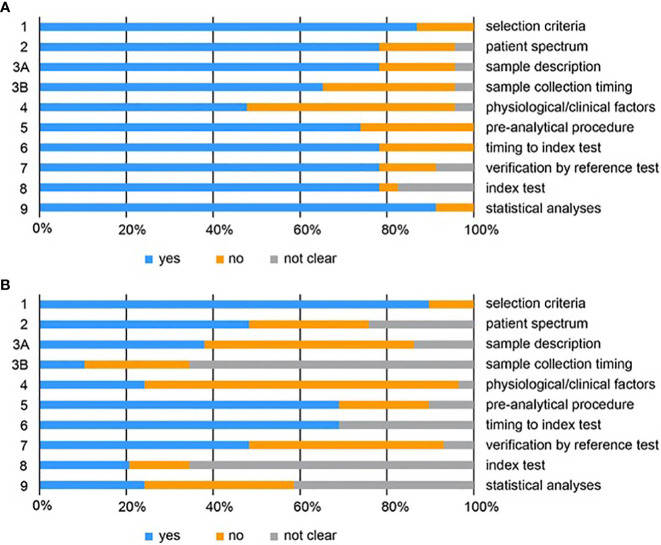
QUADOMICS scoring of all studies included for proteomics **(A)** and metabolomics **(B)**. Proportion of studies with answers “yes”, “no”, or “not clear” to each of the signalling questions. Each signalling question is numbered on the left and a short description of each question is given on the right. The detailed scoring is given in **Supplementary Tables S3** and **S4**.

The quality of the studies included was assessed systematically according to the QUADOMICS tool (**Supplementary Tables S2–S4**; **Figure 3**). We evaluated study design and pre-analytical, analytical, and post-analytical bias of all included studies. The majority of the studies described the criteria for patient selection (question 1) in appropriate detail. Approximately 50% of metabolomic and 20% of proteomic studies did not reflect the real clinical setting, because they compared patients with healthy women, who are not likely to need a diagnostic test (see discussion - question 2). The assessment of pre-analytical bias (questions 3A and 3B) revealed that only a fraction of all studies reported appropriate descriptions of the samples, including the procedures for sample collection and processing (e.g., centrifugation time, type of blood tube). Furthermore, the majority of the studies did not report any information about the time of sample collection, the time between blood draw and centrifugation, the time between sample acquisition and storage, and the number of freeze/thaw cycles. In 75% of the metabolomic and 51% of the proteomic studies sufficient information on the clinical and physiological factors that can affect -omics data was not provided (question 4; e.g., BMI, menopausal status, menstrual phase cycle, fasting status). Approximately 70% of the included studies reported detailed descriptions on sample storage and metabolite extraction (question 5). Almost all samples were stored at -80°C or in liquid nitrogen, but several studies failed to report this information. The time between the reference standard and the index test (metabolomics or proteomics) was not clear for 31% of the included metabolomic and 21% of the proteomic studies (question 6), while in 52% of the metabolomic and 22% of the proteomic studies, the verification by reference test was not performed in all patients (question 7). With respect to analytical biases, we observed that only 21% of all studies provided a detailed description of the metabolomic analysis (question 8). Most studies did not provide information on sample randomisation for MS-based metabolomics, for the use and type of quality control samples, and occasionally, important MS parameters were not given. In proteomics, 78% of all included studies described the index test in sufficient detail. Regarding post-analytical biases, we observed that 24% of the metabolomic studies described the statistical analysis in sufficient detail, while 42% of studies provided incomplete description. These studies failed to report information on missing value treatment, sample-to-sample normalization, data transformation and scaling and in one case also on model calculation and cross-validation. In proteomics, 91% of all included studies reported the statistical analysis in sufficient detail.

### 3.2 Disclosure of financial and other potential conflicts of interest

We also evaluated whether studies clearly stated the financial support, disclosed any potential conflict of interest, whether authors were affiliated to industry and whether the studies complied with the open science policy and deposited their data on public repositories (**Supplementary Table S5**). Although older studies tend not to report any information on the financial support or the presence of any conflict of interest, more recent studies provide this information. The source of funding was declared in 88% of the studies (19 out of 23 proteomic and 27 out of 29 metabolomic studies) and the presence of any potential conflict was declared in 77% of the studies (14 out of 23 proteomic and 26 out of 29 metabolomic studies). Three studies (6% of those declaring the source of funding) received industrial supports and four studies included authors affiliated to companies. Only 25% of the studies deposited the data in public repository (1 out of 23 proteomic and 12 out of 29 metabolomic studies) and four proteomic studies declare that data are available upon request.

### 3.3 Proteomics in endometrial cancer

From the systematic literature search, 72 research papers were selected based on title and abstract. From these, 19 papers were excluded because focusing on basic cell mechanisms of carcinogenesis with no further investigation on the diagnostic or prognostic potential (70–80), response to metformin (81), side effect to radiotherapy (82), racial disparities (83, 84), drug resistance (85–87), premenopausal endometrial physiology (88). Three studies were *in silico* analyses (89–91). Additionally, 11 papers (22, 92–101) were based on multi-omics approaches or focused on pan-cancer biomarkers and will be discussed in paragraph 3.4, thus resulting in 39 papers for review.

Papers that included less than 10 subjects per study group are not further discussed here (n=16; see **Supplementary Table S6** for details). This resulted in a total of 23 papers focusing on proteomic biomarkers for diagnosis, prognosis, risk stratification or classification (**Table 3** and **Supplementary Table S7**). Ten studies used blood (serum or plasma), two studies used uterine aspirate whereas eight studies used fresh frozen tissues and three used formalin-fixed-paraffin-embedded (FFPE) tissues. Various technologies were used, with the most common being 2D-DIGE/MS based methods. Although studies on tissue proteomics preceded chronologically those in body fluid, since this review focus of diagnostic/prognostic biomarkers where body fluids represent the most suitable biomaterial, we will start in the next paragraphs describing studies using body fluids for biomarker discovery.

**Table 3:** List of the 23 proteomic studies in endometrial cancer.

Study	Study aim	Samples *	Study design
Zhu, 2006 (102)	Diagnostic Biomarkers	Serum	Case - Control
Kikuchi, 2007 (103)	Diagnostic Biomarkers	Serum	Case - Control
Zhu, 2008 (104)	Diagnostic Biomarkers	Serum	Case - Control
Qiu, 2010 (105)	Diagnostic Biomarkers	Serum	Case - Control
Wang, 2011 (106)	Diagnostic Biomarkers	Serum	Cases only
Enroth, 2018 (107)	Diagnostic Biomarkers	Plasma	Case - Control
Tarney, 2019 (108)	Diagnostic Biomarkers	Serum	Nested case-control
Ura, 2021 (109)	Diagnostic Biomarkers	Serum	Case - Control
Celsi, 2022 (110)	Diagnostic Biomarkers	Serum	Case - Control
Ura, 2022 (111)	Diagnostic Biomarkers	Serum	Case - Control
Martinez-Garcia, 2016 (112)	Diagnostic Biomarkers	uterine aspirate	Case - Control
Martinez-Garcia, 2017 (113)	Diagnostic Biomarkers Prognostic Biomarkers	uterine aspirate	Case - Control
Yoshizaki, 2005 (114)	Diagnostic Biomarkers	Frozen tissue	Case - Control
DeSouza, 2007 (115)	Diagnostic Biomarkers	Frozen tissue	Case - Control
Voisin, 2011 (116)	Diagnostic, Prognostic, Therapeutic Biomarkers	Frozen tissue	Case - Control
Shan, 2016 (117)	Diagnostic Biomarkers	Frozen tissue	Cases only
Ceylan, 2020 (118)	Diagnostic Biomarkers	Frozen tissue	Case - Control
Mauland, 2017 (119)	Prognostic Biomarker associated with obesity	Frozen tissue	Cases only
Akkour, 2022 (120)	Diagnostic Biomarkers	Frozen tissue	Case - Control
Kurimchak, 2020 (121)	Prognostic Biomarkers	Frozen tissue	Cases only
DeSouza, 2010 (122)	Diagnostic Biomarkers	FFPE tissue	Case - Control
Aboulouard, 2021 (123)	Prognostic Biomarkers	FFPE tissue	Case - Control
Janacova, 2020 (124)	Prognostic Biomarkers in the tamoxifen users	FFPE tissue	Cases only

See **Supplementary Table S7** for further details. * FFPE: Formalin fixed paraffin embedded.

#### 3.3.1 Blood proteomics

Under the rationale that protein fragments/peptides are produced in the tissue microenvironment by proteolytic processes and released into the blood, the first proteomic studies based on blood (serum or plasma) were published during the first decade of 2000. Zhu and co-workers (102) performed a biomarker discovery study using SELDI-TOF-MS on 40 patients and 30 age-matched healthy controls and identified 13 m/z protein-peaks that were found in different levels between patients and healthy women. The sensitivity of each single peak ranged from 40-95%. The authors further built a decision tree-based algorithm that correctly identified 95.7% (70) of the samples (30/33 healthy women and 37/40 ECs; **Supplementary Table S7**). The same authors further improved the model using only four m/z protein peaks resulting in sensitivity and specificity of 100% and 92.3%, respectively, in the training set and 60% and 75%, respectively, in an independent validation cohort (104). The authors did not identify the proteins corresponding to the m/z spectra peaks (**Supplementary Table S7**).

A relatively large study including 199 serum samples from untreated EC patients (n=92), patients with prolapsed uterus (n=16), healthy women (n=17) (n=33), and uterine fibroids (n=74) identified 507 peaks with m/z values ranging from 2,000 to 30,000 by MALDI QTOF-MS (103). Based on predefined stringent criteria (P < 0.00001, AUC value > 0.80) three peaks were differentially abundant between the case and the control groups and showed sensitivity and specificity of 65.2% and 93.9%, respectively (**Supplementary Table S7**). Surgical stage of patients could not be discriminated by the selected m/z peaks but patients with EC and patients with uterine fibroids could be distinguished (**Supplementary Table S7**).

Qiu and co-workers (105) performed proteomics on 30 EC patients and 30 control patients on serum collected pre-operatively and identified 147 differential peaks. They further used different algorithms based on various peaks (from two to 10) and reported specificities and sensitivities up to 97% and 100%, respectively (**Supplementary Table S7**).

In another study, Wang and colleagues (106) performed a pilot study to compare the serum proteomics in patients with distinct stages of endometrial disease, from simple endometrial hyperplasia (n=6), complex hyperplasia (n=4), hyperplasia with atypia (n=4) and with early-stage EC (n=6). The authors identified 74 proteins including potential biomarkers (**Supplementary Table S7**), but the number of samples included was very limited.

A large nested case-control study aiming at identifying early detection biomarkers for EC was based on the UK Prostate, Lung, Colorectal, and Ovarian cancer screening trial (n =78,216 subjects), including 112 incident EC cases and 112 matched postmenopausal controls (108). Among cases who received an EC diagnosis less than two years after inclusion (n=31), 1,100 total proteins were identified, 565 of which were co-quantified across all patient samples and 47 proteins resulted altered compared with controls. Six candidate protein biomarkers were used to build a diagnostic algorithm with over 45% sensitivity and 96% specificity (**Supplementary Table S7**). A recent study employed PEA proteomics (PCR-based) and Olink Multiplex assays to search for candidate diagnostic biomarkers in gynaecologic malignancies, including EC (107). The authors compared malignant cases with both a group of healthy controls and with a group of women with benign tumours. The abundance of 441 unique proteins in plasma was first evaluated in a discovery phase that resulted in 16 potential protein biomarkers. The diagnostic value of nine out of these 16 proteins was validated in a replication cohort and resulted in sensitivities and specificities above 64% and 67%, respectively, to distinguish EC from healthy women or from patients with benign tumours (**Supplementary Table S7**).

Three proteomics studies using serum (109–111) were performed by an Italian group. In 2021, the authors performed a pilot study using the serum of 15 EC patients and 15 [non-cancer patients (109)] and identified 16 proteins with diagnostic potential (**Supplementary Table S7**), four of which (ITIH4, CLU, SERPIN1, and C1R) were validated by western blotting. One year later, the study was extended to a larger cohort including 60 non-EC controls and 44 EC patients (110). Proteomic analyses was performed on 10 controls and 10 EC. It is not stated in the study whether the study population and the samples used for proteomics overlaps with the previous investigation from the same team (109). The authors further validated the observed downregulation of SBSN in serum of patients by western blotting and *in silico* analysis of the TCGA database. In a subsequent study, the authors included 44 EC cases and 44 non-oncologic patients (111) - the study does not specify whether this study population overlaps with the previously studied groups (109, 110). By using PEA on two distinct protein panels (Immuno-oncology panel and Target 96 Oncology III panel), the authors identified several differentially expressed proteins and proposed different models resulting in AUCs up to 0.96 (**Supplementary Table S7**).

#### 3.3.2 Other body fluids

For diagnostics, another potentially interesting minimally invasively obtained body fluid is the uterine aspirate, which has the advantage to capture the tumour heterogeneity better than a presurgical biopsy (current standard diagnostic method). Uterine aspirate for proteomic biomarker discovery was used by Martinez-Garcia and collaborators (112, 113). Since the LC-PRM targeted proteomics technique allows the quantification of a limited number of predefined proteins, the authors adopted a sequential workflow: 506 candidate biomarkers were first extracted from a literature search. Subsequently, the authors determined the presence of these biomarkers in uterine aspirates by LC-MS/MS and confirmed the presence of 158 proteins. After method optimisation, a list of 52 candidate biomarkers was selected for PRM design/development and 26 proteins were differentially expressed between cases and controls (112). The same set of 52 proteins was subsequently tested on an independent prospective cohort of 116 women entering the EC diagnostic workup due to EC suspicion (113). A diagnosis of EC was confirmed in 69 women and 28 proteins elevated in EC *versus* controls had an AUC >0.75. Various tests and combinations of the five best individual biomarkers were assessed, resulting in diagnostic and prognostic models with sensitivities and specificities above 89% and 83%, respectively (**Table 3**).

#### 3.3.3 Tissue proteomics

##### 3.3.3.1 Frozen tissue

Pioneering studies were conducted as early as 2005 using iTRAQ. After determining the feasibility and comparing the performance of iTRAQ and cICAT for proteomics (authors used less than 10 samples per group; **Supplementary Table S6**, (125), the authors used iTRAQ to analyse 40 frozen tissue samples including proliferative, secretory endometrium and EC (115). Over 1,000 proteins were identified among which six candidate markers (PK, PIGR, CPN10, MIF, AAT, CKB, and TAGLN) were confirmed as differentially expressed from their previous pilot investigation (125). Fourteen proteins were selected for further analyses and after assessing the associations of each individual protein with malignant or benign status using the two-sample t-test (p<0.005), four proteins (PK, CPN10, AAT and CKB) were selected to build a prediction model. Although these proteins used as single markers reached maximum AUC of 0.95 (sensitivity: 85%; specificity: 90%; PV: 87%; PPV: 89%), the use of three markers (AAT, PK and CPN10) resulted in improved performance and an AUC of 0.96 (**Table 3**). The validity of these biomarkers was further confirmed by two-thirds/one-third cross-validation, and also by using dot-blot and IHC on a panel of independent samples (115). In a subsequent study, the authors verified five of the identified markers (CPN10, S100A8, PIGR, PK-M2 and AAT) and one additional marker (TIMP-1) by IHC on a tissue microarray (TMA), including 148 samples (two simple hyperplasia; eight complex hyperplasia; 39 endometrioid EC; 13 serous papillary/clear cell or Type II EC; one carcinosarcoma: 85 benign endometrium samples of which 25 proliferative, 25 secretory, 25 atrophic, and 10 menstrual). They further showed that CPN10 and PK-M2 could distinguish hyperplasia and EC cases *versus* controls (sensitivity and specificity of 77% and 87%, respectively), whereas the combination of AAT, CPN10, and PK-M2 resulted in sensitivity and specificity of 85% and 93%, respectively, in distinguishing EC *versus* control patients (126). The same authors subsequently performed a pilot study on 10 EC patients and 10 control patients using a novel strategy (drill-down coupled to iTRAQ) to improve their ability to detect novel proteins and identified 1,529 proteins, among which 40 candidate biomarkers. The PPV and AUC of these proteins used as single diagnostic biomarkers ranged between 62%-100% and 0.60-1.00, respectively (**Supplementary Table S7**) (116).

In parallel to these studies based on iTRAQ, the first studies analysing fresh/frozen EC tissues using 2D gel separation followed by MS were published in 2005. By applying SELDI-TOF-MS to 19 cases of EC and 20 control patients, the authors identified one peak (m/z 9,600) consistently upregulated and a second peak (m/z 11,300) consistently downregulated in case group versus control group (114).

Additional biomarker discovery studies were published in subsequent years, with most of them being pilot or feasibility in nature and including less than 40 samples. Shan and co-workers (117) compared EC *versus* adjacent normal tissue in 10 cases with iTRAQ-based proteomics and identified 1,266 proteins, 103 of which were upregulated and 30 downregulated in cancer *versus* control tissue (**Supplementary Table S7**). Results were confirmed by western blotting, qRT-PCR and functional studies using cell lines. Ceylan and co-workers (118) also performed a diagnostic biomarker discovery study based on 2D-DIGE-MALDI-TOF and compared controls (pre- and post-menopausal women), hyperplasia and EC. Several proteins were differentially expressed between controls and EC, controls and hyperplasia (**Supplementary Table S7**), or were associated with advanced-stage disease (CAH1, PPIB, K2C8, and UAP56).

Mauland and colleagues (119) explored the levels of 163 proteins using RPPA in relation to prognosis and obesity. The authors used patient cohorts from different geographical regions: a group of samples collected in Norway in two different periods served as training (n=272 collected between 2001-2013) and validation (n=68 collected between 2011-2015) cohorts and a third cohort collected in Texas (USA) was used as extra validation (n=178 collected between 2000-2009). Beside correlation with BMI, several proteins were associated with patient prognosis, including proteins indicative of a low PI3K activation in non-obese early-stage ER-positive tumours. Data was further validated by RNA (correlation) and IHC (**Table 3**). Akkour and colleagues (120) used 2D-DIGE to analyse tissues from patients with hyperplasia (n=12), EC (n=12) and age-matched control patients (n=12) and identified 87 differentially expressed proteins (26 between controls and hyperplasia and 32 between EC and hyperplasia; **Table 3**). Further modelling was not performed. In a recent study, Kurimchak et al. (121) used an innovative approach based on Multiplexed Inhibitor Beads (MIB) and MS to chart the kinase network in EC (n=20) and adjacent normal tissues (n=16). The MIB binding value was measured for 347 kinases, 300 of which were quantitated by both LFQ and s-SILAC, whereas 37 and 10 by LFQ and s-SILAC, respectively. These analyses showed that SRPK1 was overexpressed in cancer tissue (**Table 3**). IHC on TMAs (39 serous and 18 endometrioid and 12 normal endometrial tissues), functional/loss of function studies *in vitro* and the TGCA and CPTAC datasets confirmed that SRPK1 is associated with EC and with poor patient survival.

##### 3.3.3.2 Formalin-Fixed-Paraffin-Embedded (FFPE) tissue

A number of studies investigated the potential use of FFPE material for proteomics (**Supplementary Table 6**), but only three of them met our selection criteria (**Table 3**; **Supplementary Table S**). DeSouza and colleagues confirmed the feasibility of mTRAQ targeted proteomics using FFPE tissues (122). The authors laser-capture-microdissected tissue and examined the tissue of interest from 10 ECs and 15 proliferative endometrium samples and detected 13 out of the 17 targeted proteins across 12 samples (**Table 3**; **Supplementary Table 7**).

Janacova and colleagues (124) explored archival material from 36 EC patients, 15 of whom had received tamoxifen adjuvant treatment for breast cancer, whereas 21 were never exposed to tamoxifen previously. The authors explored with LC−MS/MS in SWATH-MS mode 34 tumour samples (each from one subject) and 11 myometrial tissues adjacent to the tumours. The proteomic approach targeted over 1,100 different proteins, of which over 900 were consistently identified. The authors compared clinical features in the tamoxifen *versus* tamoxifen naïve patients and identified six upregulated and 22 downregulated proteins. The expression of CAPS and STMN1 was confirmed with IHC and STMN1 was also associated with poor patient prognosis (**Table 3**). Using a very innovative approach (123), Aboulouard and coworkers compared the proteome profile in EC and sentinel-lymph-node SLN tissues (**Table 3**; **Supplementary Table 7**). Regions of interest were first microdissected, then analysed with NanoLC-ESI-MS and a number of potential biomarkers indicative of lymph node disease were identified.

### 3.4 Metabolomics in endometrial cancer

Our literature search identified 29 studies using metabolomics in EC (**Table 4** and **Supplementary Table S8**), with the majority evaluating the metabolic profiles in blood samples (10 serum, seven plasma, one serum and plasma, one dried blood). Seven studies focused on endometrial tissue samples, one on cervical lavage, one on endometrial brushing and one study on urine samples. Most studies aimed to identify diagnostic and/or prognostic biomarkers, better understanding the mechanisms of carcinogenesis (studies in tissue samples), and also to determine associations between metabolic profiles and EC (131, 132). Plasma, serum and urine represent appropriate sources for discovery of diagnostic/prognostic biomarkers (155). However, also metabolic profiles of cervical lavage, brushing endometrial samples, and tissue samples (if obtained as pre-surgical biopsy) may be of clinical relevance. Non-targeted metabolomics was more commonly applied (20 studies) as compared to targeted metabolomics (10 studies). Only six studies used NMR analysis, and there was one study that combined NMR with the most commonly used LC-MS/MS (138). The majority of the targeted metabolomic studies focused on lipids and amino acids.

**Table 4:** List of the 29 metabolomic studies in endometrial cancer.

Study	Study aim	Samples	Study design
Ihata, 2014 (127)	Diagnostic Biomarkers	Plasma	Case-control
Knific, 2018 (128)	Diagnostic Biomarkers Prognostic Biomarkers	Plasma	Case-control
Strand, 2019 (129)	Prognostic Biomarkers	Plasma	Cases only
Njoku, 2021 (130)	Diagnostic Biomarkers Prognostic Biomarkers	Plasma	Case-control
Kliemann, 2021 (131)	Association	Plasma & serum	Nested case-control
Dossus, 2021 (132)	Association	Plasma	Nested case-control
Breeur, 2022 (133)	Association	Plasma & serum	Case-control study
Audet-Delage, 2018 (134)	Diagnostic Biomarkers Prognostic Biomarkers	Serum	Case-control
Audet-Delage, 2018 (135)	Diagnostic Biomarkers Prognostic Biomarkers	Serum	Case-control
Troisi, 2018 (136)	Diagnostic Biomarkers Prognostic Biomarkers	Serum	Case-control
Shi, 2018 (137)	Exploratory	Serum	Case-control
Bahado-Singh, 2017 (138)	Diagnostic Biomarkers	Serum	Case –control
Lunde, 2020 (139)	Exploratory	Serum	Cases only
Kozar, 2021 (140)	Exploratory	Serum	Prospective observational study
Gu, 2021 (141)	Diagnostic Biomarkers Prognostic Biomarkers	Serum	Case-control
Yan, 2022 (142)	Diagnostic Biomarkers Prognostic Biomarkers	Serum	Case-control
Schuhn, 2022 (143)	Diagnostic Biomarkers	Serum	Case-control
Troisi, 2020 (144)	Diagnostic Biomarkers	Dried blood samples	Multicenter prospective cohort study
Shao, 2016 (145)	Diagnostic Biomarkers	Urine	Case-control
Cheng, 2019 (146)	Diagnostic Biomarkers	Cervicovaginal fluid	Case-control
Jove, 2016 (147)	Diagnostic Biomarkers	Tissue	Case-control
Altadill, 2017 (148)	Diagnostic Biomarkers	Tissue	Case-control
Trousil, 2014 (149)	Diagnostic Biomarkers	Tissue	Case-control
Cummings, 2019 (150)	Diagnostic Biomarkers	Tissue	Case-control
Skorupa, 2021 (151)	Diagnostic Biomarkers	Tissue	Case-control
Arda Düz, 2022 (152)	Diagnostic Biomarkers	Tissue	Case-control
Gatius, 2022 (153)	Diagnostic Biomarkers	Tissue from Biobank	Cases only
Shafiee, 2020 (154)	Diagnostic Biomarkers	Plasma & tissue	Cros-sectional study
Yi, 2022 (101)	Diagnostic Biomarkers	Tissue & Urine	Case-control

See **Supplementary Table S8** for further details.

#### 3.4.1 Blood metabolomics

Metabolomic studies on serum samples from EC patients have been performed from 2017 and identified a series of metabolites in differential concentrations between study groups (**Supplementary Table S8**). Audet-Delage and co-workers (134) reported that the levels of 115 acylcholines, monoacylglycerols, and acylcarnitines were increased while the levels of 22 free fatty acids were decreased in 26 postmenopausal EC patients (type I and II, recurrent and non-recurrent) *versus* 18 patients with benign conditions. The authors identified a series of metabolites specific for recurrent EC, where bile acids were increased in type I and sphingolipids in type II recurrent EC. The authors constructed a diagnostic model (including the levels of spermine, isovalerate, glycylvaline and gamma-glutamyl-2-aminobutyrate) with an AUC of 0.92 and a prognostic model (including 2-oleoylglycerol and TAG 42.2-GA12:0) that separated between recurrent and non-recurrent EC with an AUC of 0.90. Troisi and colleagues (136) used a GC-MS approach and determined the metabolic profiles in 118 EC patients and 130 healthy women and control patients. Using several machine learning approaches and distinct patient cohorts, they constructed and validated a diagnostic model (EC *versus* healthy women) with accuracy of 0.99 and a prognostic model (type I/type II) with accuracy of 0.93. The first was based on increased levels of lactic acid, homocysteine, 3-hydroxybutyrate, and decreased levels of linoleic acid, stearic acid, myristic acid, threonine, valine and progesterone, whereas the latter on increased levels of progesterone and decreased levels of lactic acid, cystine, serine, malate, glutamate and homo-cysteine. Bahado-Singh and co-workers (138) performed NMR analysis in 56 stage I-IV EC patients and 60 healthy women, divided in discovery (33 ECs and 36 healthy women) and validation phase (23 EC and 24 healthy women) and constructed several diagnostic logistic regression models based on lipid levels with an AUC above 0.8. The highest AUC (0.83) was reported for the combination of C14:2, PCae C38:1 and 3-hydroxybutyric acid. This model separated also between stage I-II EC and healthy women (AUC = 0.82). An exploratory MS analysis by Kozar et al. in 15 EC and 21 control patients (140) reported a Random Forest model including Cer 34:1;2, Cer 40:1;2, AC 16:1-OH and 1-methyladenosine with AUC of 0.92, but reported no validation. Yan et al. performed a MS-based study (142) which included 23 EC patients, 30 healthy women, 30 patients with endometrial polyps and 12 patients with endometrial hyperplasia in the discovery phase and 50 EC patients (stage I-IV) and 195 healthy women, 171 polyps and 40 hyperplasia patients in validation phase. Their logistic regression models for separation between EC and endometrial polyps included 6-keto PGF1α, PA(37:4), LysoPC (20:1) and PS (36:0) and showed good characteristics with AUC > 0.90. A recent MS/MS targeted metabolomic study by Schuhn et al. performed in 20 EC patients, 157 healthy women and 14 control patients (143) reported that individual metabolites (carnitines and amino acids) allow stratification between EC and healthy women, and EC and control patients, with AUCs of 0.82 and 0.85 for malonylcarnitine and threonine, respectively (**Supplementary Table S8**). The study by Lunde et al. (139) performed NMR analysis in serum samples from 78 EC patients stored in Danish Cancer Biobank to determine metabolic profiles that allow identification of patients with chronic pelvic pain after hysterectomy. Using different machine learning approaches on metabolites with different levels in the two groups (**Supplementary Table S8**), several models were built, with the best diagnostic characteristics (AUC of 0.87) seen for linear support vector model.

Due to potential variability in the composition of serum, plasma represents the preferred source for biomarker discovery. However, so far a minority of the studies on blood metabolomics were performed using plasma. The first study by Ihata et al. (127) used MS to analyse plasma from 80 EC patients (stages I-IV), 122 patients with benign gynaecological diseases and 240 healthy women using training (40 EC and 120 healthy women) and validation sets (40 EC and 120 healthy women and 122 control patients). The authors built logistic regression diagnostic models based on panels of amino acids (histidine, isoleucine, valine and proline) that separated EC from healthy women (AUC > 0.91) and EC from control patients (AUC = 0.83; **Supplementary Table S8**). In a study by Knific et al. (128), 61 EC patients and 65 patients with benign uterine conditions were included. By employing LC-MS/MS analysis in training and test sets, the authors constructed diagnostic logistic regression models to separate EC from controls (AUC = 0.84) and prognostic models that allowed stratification of patients with lymphovascular invasion (LVI; AUC = 0.94) and myometrial invasion (AUC = 0.86). These were the first diagnostic and prognostic models of EC that included metabolite ratios. Strand et al. (129) used the same methodological approach but focused on prognostic biomarkers to identify metabolic differences between 20 EC patients with long *versus* 20 EC patients with short survival, where patients were matched for stage, grade, age, and BMI. Using Partial Least-Squares Discriminant Analysis (PLS-DA), three models with AUC up to 0.96 were constructed but none has been validated yet (**Supplementary Table S8**). Another MS-based study in plasma samples (130) focused on diagnosis of EC in obese patients (BMI > 30) and included 67 EC patients and 69 control patients (test and training sets). RF algorithms including 20 metabolites separated all EC patients from controls (AUC = 0.95) and showed even better characteristics for separation of stage I EC from control patients (AUC = 0.98). Individual metabolites showed potential as prognostic biomarkers and separated EC patients with/without LVI (AUC = 0.83; **Supplementary Table S8**). Other studies on serum/plasma metabolome in EC patients reported only different levels of metabolites in EC patients (133, 135, 137, 141) and associations of individual metabolites with EC (131, 132).

A well-designed GC-MS discovery study analysed dried blood samples analysed 50 postmenopausal EC patients and 70 patients without EC and validated prospectively the results among 1,430 postmenopausal women including 16 incident EC patients (**Supplementary Table S8**). Their ensemble machine-learning algorithm included 10 different classification models with accuracy of 99.9% (144). Among studies reporting serum and/or plasma metabolic profiles in EC patients, only few diagnostic/prognostic models have been validated in large multicenter studies, and the majority still awaits appropriate validation.

#### 3.4.2 Metabolomics in other physiological fluids

Only two studies searched for biomarkers of endometrial cancer in urine samples (**Supplementary Table S8**). Shao et al. (145) used nontargeted metabolomics to determine differences in urine metabolic profiles from 25 EC patients, 25 healthy women and 10 endometrial hyperplasia patients and constructed PLS-DA and Support Vector Machine models, but provided no diagnostic characteristics. Yi et al. (101) analysed urine, tissue samples, and brushing endometrial samples and identified 285 metabolites in differential levels in urine samples from 10 EC patients compared with 10 control patients. PLS-DA based on the top 100 metabolites showed an AUC of 0.81. The cervicovaginal fluid from 21 EC patients and 33 non-EC controls was analysed by NMR using training and test sets (146). The levels of 29 metabolites differed between groups and RF and SVM models with accuracy up to 0.78 were constructed (**Supplementary Table S8**). These studies in urine samples and cervicovaginal fluid included a small number of samples thus future attempts for biomarker discovery should include respective metabolomics profiles from larger group of EC and control patients.

#### 3.4.3 Tissue metabolomics

Seven studies explored the metabolic profiles in EC tissue. The first study was published in 2014 (149) and included 10 ECs and 10 control patients. NMR analysis revealed deviated concentration of of several amino acids, phosphocholine, glutathione, scyllo-inositol, myo-inositol, and inosine/adenosine in EC tissue (**Supplementary Table S8**) and the authors built a PLS-DA model with an AUC of 0.99. Arda Düz et al. (152) employed NMR to analyse tissues from 17 ECs and 18 control patients, and reported a number of candidate metabolite biomarkers e.g., lactate, alanina, phenylalanine and ratios glutamate/glutamine/methionine and leucine/isoleucine with AUCs up to 0.88 (**Supplementary Table S8**). A recent non-targeted NMR analysis on 64 EC tissue (patients with different grades of disease) and 10 tissues from patients with benign uterine diseases, identified using OPLS-DA the levels of a number of metabolites differentiating the patient groups (151). The concentrations of dimethylsulfone and phosphocholine were higher whereas the concentrations of glycerophosphocholine and glutamine were lower in low grade EC. In grade 1/2 EC, the levels of myoinositol were decreased and in grade 3 there were higher levels of 3-hydroxybutyrate, alanine, and betaine. The models constructed based on individual metabolites allowed separation between different grades of tumours with AUCs above 0.90 (**Supplementary Table S8**).

Other studies that investigated the metabolic profiles in cancerous tissues contributed mainly to a better understanding of the pathophysiology of EC, as these studies reported differential metabolites and dysregulated pathways in EC. Jove et al. (147) analysed 27 EC tissue samples and 15 normal endometrium samples by MS/MS and identified 44 differential metabolites including increased levels of stearamide, monoolein, hypoxanthine, 1,2-dihexadecanoyl-sn-glycerol (**Supplementary Table S8**). Comparison of cancer tissue of different grades identified 26 metabolites with increased levels of taurine and erythriol and decreased levels of oleamide. Importantly, this nontargeted metabolomic study used a novel approach as the authors examined the differences between surface EC and the myometrial invasion front and reported 104 differential metabolites (147). Altadil et al. (148) used non-targeted MS/MS to analyse 39 EC tissues and 17 control samples from postmenopausal women with stage I-III EC and benign diseases, respectively. Eighty metabolites, with 42 exhibiting differential levels, were identified with increased levels of taurine and erythriol and decreased levels of oleamide (**Supplementary Table S8**). Specific metabolites had different levels between cancers and controls (glutamate-phenylalanine-arginine-tryptophan, palmic amide, stearamide, oleamide, 2-phosphatidylserine, phosphatidylglycerol, inosine, and picolinic acid) or between stage I/II and stage III disease (phosphatidylcholines, phosphatidylethanolamines, and arachidonic acid; (**Supplementary Table S8**). Cummings et al. (150) performed a targeted metabolomic study on 108 cancer tissues, 53 samples of normal endometrium, 33 atrophic endometrium and 31 samples of atypical hyperplasia. The authors showed decreased concentrations of a number of metabolites including 13,14-dihydro-15-keto PGE_2_ in type 1 and 2 EC versus normal endometrium and 12-HETE in EC type 2 versus type 1; **Supplementary Table S8**). Shafiee et al. (154) focused on the pathophysiology of EC and compared 34 cancerous tissues with 34 control endometrial tissues from patients with polycystic ovarian syndrome (PCOS). Their nontargeted MS-based analysis revealed changes mainly among lipids. Yi et al. (101) performed nontargeted metabolomics in urine, intrauterine brushing, and tissues from 24 EC patients and 18 control patients where PLS-DA identified 74 metabolites of which 47 were found in higher levels and 27 in lower levels. Comparison of metabolic profiles in tissue samples to urine and brushing samples showed that 49 of 74 metabolites were also detected in urine samples and 21 of 74 metabolites in intrauterine brushing samples, which supports the potential of urine metabolomics profiles for non-invasive diagnostic/prognosis (**Supplementary Table S8**). A recent study explored the metabolic profiles in biobanked tissue samples from endometrioid (n=20) and serous EC (n=11) (153). Using non-targeted MS analysis, 232 metabolic differences could be characterised (**Supplementary Table S8**).

Three metabolomic studies using tissue samples (149, 151, 152) identified individual metabolites and constructed diagnostic models with promising AUC values; however, these studies included very limited number of patients.

### 3.5 Combined metabolomics/proteomics

There was only one study that employed a combined omics approach (101) and performed nontargeted metabolomics on 24 cancer and 20 control tissue samples (**Supplementary Table S7**) and also nontargeted proteomics on a subset of 12 cancer and 9 control tissue samples by LC-MS/MS. The authors identified 1,445 proteins significantly up- or down-regulated in the EC group compared with the control group (adj. p<0.05, FC<1.5). To further characterise any relation between the metabolic and proteomic profiles, the authors performed network analysis that showed 28 metabolites and 135 proteins with 212 connections. Glutamine, dopamine, noradrenaline, adenosine-5-monophospate, and guanosine-5’-monophosphate were the major centres of sub-networks showing differences in amino acid and nucleotide metabolism.

### 3.6 Additional multi-omics or pan-cancer studies

A number of studies explored the proteome in patients with EC, but such analyses were part of a larger multi-omics approach, or part of systems-biology/pan-cancer approaches to study human disease or to establish databases and repositories. Most of these studies did not use proteomics for biomarker discovery, but as tools to understand the pathophysiological processes.

Five studies aiming at improved patient classification were performed within the TCGA consortium and used RPPA as proteomic method (22, 92, 94, 95, 99). Two studies demonstrated the utility of functional proteomics based on RPPA next to genomics and transcriptomics (92) and further created the bioinformatic resource ‘The Cancer Proteome Atlas’ (TCPA; (94)). The study of Kandoth and co-workers (13) explored a cohort of 373 EC patients, including 307 endometrioid and 53 serous or mixed histology cases to assess somatic mutations, copy number alterations, RNA expression, protein expression, DNA methylation and micro-RNA expression. With regard to proteomics, 293 samples were analysed by RPPA and several differentially expressed proteins were associated with other specific molecular tumour features (22). One of the latter two TCGA studies did not use proteomics (99), whereas the other study (95) explored 57 carcinosarcomas and -also in this case-, protein analyses were used to confirm other features identified with the molecular analyses (like EMT transitions, PI3K/AKT pathway activation, low steroid hormone receptor signalling). Similar to the TCPA (initiated within TCGA), the Clinical Proteomic Tumor Analysis Consortium (CTPAC) generated large proteomic datasets across various tumour types, and further characterised the proteogenomic landscape in EC (100). A pan-cancer study (98) aimed to characterise the actionable mutations across different solid tumours (including EC) and used RPPA to demonstrate PI3K and MAPK signalling pathways, whereas one study was focussed on human diseases other than cancer (97).

## 4 Discussion

In this study, we systematically reviewed all papers that explored the proteome or the metabolome in search for candidate biomarkers for prognostic or diagnostic purposes in EC. After screening the retrieved publications, we included 23 studies on proteomics (serum, plasma, uterine aspirate or tissue) and 29 studies on metabolomics (serum, plasma, urine, intra-uterine brushing, dried blood, cervicovaginal fluid or tissue).

Proteomic studies on body fluids and tissues (first fresh frozen then FFPE) have been published from second half of 2000. Initial studies were pilot in nature, and enrolled only a few patients (**Supplementary Table S6**). The first metabolomic studies were published one decade later, and study populations were in general larger than those used for proteomics. Seven out of 23 proteomic and 14 out of 19 metabolomic studies reported the performance of the models as AUCs, and several candidate biomarkers show great potential with AUC values above 0.8. However, the majority of the reported proteins or metabolites and corresponding models represent biomarker candidates that still require validation. The models developed need evaluation of their statistical performance by splitting the data into training and test sets (so called “statistical validation”) and further experimental validation on independent cohort is essential (156).

With regard to statistical validation, this was performed by two proteomic studies (two-thirds/one-third cross-validation (115); leave-one-out-cross-validation (113) and four metabolomic studies (127, 128, 136, 142). Data on differentially expressed proteins was also confirmed experimentally using alternative methodologies like dot-blot (115), IHC (119, 121), RNA/qRT-PCR (117, 119), western blotting (110, 117), *in vitro* functional studies (117, 121) or using existing databases and repositories (119, 121).

In the context of proteomics, four studies only validated their data using independent sample cohorts (104, 107, 113, 119), and one study (115), validated their proteomic-based model in a subsequent publication using TMAs (126). Three metabolomic studies performed similar validations in independent cohorts (138, 142, 144), and one study in particular successfully validated the metabolic profiles identified in serum samples (136) also using dried blood and reported excellent diagnostic characteristics (144). For validation, the authors adopted a prospective design on a very large study cohort (over 1,000 subjects), therefore, candidate biomarkers identified in this study bear great potential for translation into clinical practice. Of interest, these biomarkers include steroids, which were also selected as candidate molecules in other studies (135, 157)

A caveat in a number of the included studies on proteomics (102, 104, 108) or metabolomics (136, 138, 142, 143) is the use of healthy (not age/comorbidity matched) women, likely resulting in an overestimated diagnostic accuracy. Also, if pre-menopausal controls are included, this may induce biases as EC is predominantly a postmenopausal disease (114–116, 122, 147, 149, 154). Additionally, biomarker discovery preferably includes a relevant population needing diagnostic tests such as women with postmenopausal uterine bleeding, or high-risk women, e.g., patients treated with tamoxifen or with Lynch syndrome, but also women with PCOS and obesity. Some proteomic studies focussed on these target groups, like obese subjects (119), or women with previous exposure to tamoxifen (124). One metabolomic study was performed in an obese population, and reported a Random Forest-based diagnostic model combining the top 10 performing metabolites that stratified stage I EC from other obese patients with an AUC of 0.98 (130), whereas the second metabolomic study included patients with PCOS (154).

A final relevant confounder is the ethnic background, known to affect the proteome profiles (83, 84). However, only one study used a cohort from a different geographical background, although still Caucasian to validate their data (119).

In a diagnostic/preoperative workup, ideal biomarkers should be present in easily and minimally invasively obtained body specimens. The proteomic studies included in this review predominantly used blood (serum of plasma) or uterine aspirate. The pilot studies using urine, although possibly the ideal body fluid for biomarker detection, were too small to be included (**Supplementary Table S6**). Also in the context of metabolomics, only a few studies were performed on physiological fluids other than blood. One study explored cervicovaginal fluid (146) and two studies used urine samples, both of which have important limitations as they either used healthy women as controls (145) or included a few samples only (10 EC patients and 10 control patients) (101). Despite these limitations, these studies are also promising and future attempts for biomarker discovery in urine or cervicovaginal fluids using larger group of EC and control patients are warranted.

Importantly, although research groups were often able to validate their own candidate biomarkers in subsequent studies, rarely data was confirmed by independent authors/researchers, most probably due to methodological issues and also the abovementioned biases associated with geographical locations/ethnicity and lifestyles. Of note, there were candidate biomarkers that were validated in independent studies, and these represent highly promising molecules. Besides lipids, phospholipids and steroids, candidate proteins were reported as well. ANXA1 is described upregulated by three independent groups in EC tissues, uterine aspirates, and lymph nodes contaminated with EC cells (112, 113, 118, 123). ANXA1, annexin A1, plays an important role in immunity and inflammation and is associated with various diseases and cancers (158). HSPB1 was found upregulated in EC tissues and uterine aspirates by two teams (112, 113, 118). The *HSPB1* gene encodes for Heat Shock Protein Family B (Small) Member 1, a protein that is associated with gynaecological cancers (159). In addition, SERPINC1, APOA4, APOE and ITIH4 are described deviated in the serum of hyperplasia or cancer patients by two teams (106, 109). In metabolomic studies, both molecule levels but also the ratio between levels of molecules proved to be good biomarkers, and a number of studies included metabolite ratios in modelling/analyses (128, 130, 132, 148, 152).

Overall, the major limitations of the studies published up to data are: i) the use of small study cohorts; ii) the diagnostic or prognostic accuracy was seldom compared with other known biomarkers or reference molecules (e.g., CA-125). iii) the 95% confidence intervals for AUC values, sensitivity and specificity were rarely reported; iv) as outlined above, validation in independent cohorts was done by a few studies only; specifically in metabolomic studies, validation using other technologies was never performed.

### 4.1 Strengths and limitations of the present study

The limitations reported above related to the papers retrieved and reviewed are reflected also in the present study, i.e., small study cohorts, potenial pre-analytical and analytical bias, potential bias due to ethnic background, lifestyle; lack of validations; no comparison with a reference or gold standard. This, in combination with the heterogeneity in study designs and in the technologies adopted precluded us making any meta-analyses of identified candidate biomarkers, whose potential can be assessed at a qualitative level only. This implies the impossibility to make any clinically relevant recommendation or conclusion at this moment. Nevertheless, the strength of the present study is that, due to the rigorous systematic approach we adopted, it offers a balanced and realistic view of the potential of these technologies for the future. It sets the milestones in proteomic and metabolomic biomarker discovery research, and indicates the path to follow in the future (see paragraph 4.2. Recommendations for further biomarker discovery).

It should be also noted that recently systematic reviews focused on metabolomic and/or proteomic biomarkers for diagnosis of EC (160), and liquid biomarkers for diagnosis of EC (161). However, our systematic review has additional unique strengths: we did not limit our analyses on one biospecimen only, but focused on metabolomics and proteomics in different biospecimens; we rigorously assessed the study quality using QUADOMICS and analysed additional potential conflicts of interest. The study quality was also assessed by Karkia and co-workers (161), however, these authors included only studies published in the two years prior to the publication, whereas we did not set a time limit for publication. Additionally, we used strict inclusion and exclusion criteria, which were defined and deposited in the PROSPERO repository prior to the start of our work. We finally provide comprehensive tables with all available diagnostic accuracy data (AUC, sensitivity, specificity).

### 4.2 Recommendations for further biomarker discovery

As thoroughly discussed, body fluids and liquid biopsies represent the most suitable material for diagnostic and prognostic biomarkers (162, 163), as they capture the disease heterogeneity better than a biopsy and they are non- or minimally invasive, thus create less anxiety in patients. In the context of the most appropriate body material, plasma preparation is less prone to technical (pre-analytical) biases than serum. Therefore, plasma represents the preferred source at least for the discovery phase of non-invasive diagnostic/prognostic biomarkers. Urine also represents an important clinical sample for non-invasive diagnostics (155), calling for further biomarker discovery studies. However, urine poses a challenge for biobanking, as large sample volumes are needed for analyses (this applies not only if 24-hour urine needs to be collected, but also for morning urine, which is common in biomarker discovery studies).

In terms of methodology, statistical and experimental validation in independent cohorts should be an intrinsic part of biomarker discovery studies, and inclusion of study populations with distinct lifestyles, geographical regions of origin and ethnic backgrounds is essential to identify candidate biomarkers truly associated with diseases. Strict standard operation procedures for sample collection and processing should be prepared by experts and rigorously followed (164). The importance of adhering to quality standards is also emphasized in a recent narrative review on biomarker discovery in EC (165).

## 5 Conclusions and future prospects

Clinically, there is a great need for non/minimally invasive biomarkers of EC that could serve as replacement test for endometrial biopsy or a triage test to select patients for further invasive diagnosis. Tissue biomarkers are also needed to allow preoperative stratification of patients and further individualised treatment. Recent advances in analytical technologies and computational approaches that can handle increasingly larger numbers of features offer unprecedented potentials to develop diagnostic and prognostic tools. The studies performed so far were in most cases pilot or explorative in nature, and heterogeneous in terms of study design, technology, and methodologies. These aspects need harmonisation for the future, and the study quality should be scrupulously monitored by journals, reviewers, and stakeholders in order to ensure translationability of the discoveries.

## BIOENDOCAR

Members of the consortium besides the authors: Špela Smrkolj, Luka Roškar, Boštjan Pirš, Department of Gynecology, University Medical Centre Ljubljana, Slovenia; Aneta Adamiak-Godlewska, Sara Wawrysiuk and Ola Kaminska, Department of Gynaecology, Lublin Medical University, Poland. External collaborators: Jure Knez, Monika Sobočan and Iztok Takač, University Medical Centre Maribor, Slovenia; Fabio Barra, Department of Obstetrics and Gynaecology, IRCCS Ospedale Sacro Cuore - Don Calabria, Genoa, Italy; Simone Ferrero, Academic Unit of Obstetrics and Gynaecology, IRCCS Ospedale Policlinico San Martino; Vit Weinberger and Petra Vinklerova, Department of Gynecology and Obstetrics, University Hospital Brno, Czechia.

## Author contributions

AR, TR, HW, AS, CL, CS, AG, JA, DF, and JT contributed to design the study, AR and TR to the literature search, AR, TR, HW, AS, CL, AG, DF and JT to writing the manuscript and issuing the figures. All authors contributed to the article and approved the submitted version.

## Glossary

**Table:**

AUC	area under the curve
BMI	body mass index
CPTAC	Clinical Proteomic Tumor Analysis Consortium
Da	Dalton
DFS	disease-free survival
DIGE	difference gel electrophoresis
EC	Endometrial cancer
ELISA	enzyme-linked immunosorbent assay
ER	estrogen receptor
ESI	electrospray ionization
FFPE	formalin-fixed-paraffin-embedded
FIGO	International federation of gynaecologic oncology
GC	Gas chromatography
HER2/Neu	Human Epidermal growth factor Receptor 2
HR	hazard ratios
iCAT	Isotope-coded affinity tag
IHC	immunohistochemistry
iTRAQ	Isobaric tags for relative and absolute quantitation
LC	Liquid chromatography
LFQ	Label free quantification (LFQ)
LVI	lymphovascular invasion
MALDI	matrix-assisted laser desorption/ionization
MIB	Multiplexed Inhibitor Beads
MRM	multiple-reaction monitoring
MS	mass spectrometry
MSI	mass spectrometry imaging
NMR	nuclear magnetic resonance spectroscopy
NPV	negative predictive value
OS	overall survival
PAGE	polyacrylamide gel electrophoresis
PCOS	Poly Cystic Ovarian Syndrome
PEA	proximity extension assay
PLS-DA	Partial Least-Squares Discriminant Analysis
PPV	positive predictive value
PR	progesterone receptor
PRM	parallel reaction monitoring
PTEN	Phosphatase and tensin homolog
RPPA	reverse phase protein microarray
SD	Standard Deviation
SDS	sodium dodecyl sulphate
SELDI	surface-enhanced laser desorption/ionization
SILAC	Stable isotope labelling by amino acids in cell culture
SLN	sentinel-lymph-node
SRM	Selected-reaction monitoring
TCGA	The Cancer Genome Atlas
TCGA	The Cancer Proteome Atlas
TMA	tissue microarray
TOF	time-of-flight
