# Supplementary material for: Endometrial cancer diagnostic and prognostic algorithms based on proteomics, metabolomics, and clinical data: a systematic review
Source: Front Oncol. 2023 Apr 6;13:1120178. doi: 10.3389/fonc.2023.1120178 (PMC10118013; doi:10.3389/fonc.2023.1120178)
Supplement: Supplementary file 1 [file Table_1.docx]

**Endometrial cancer diagnostic and prognostic algorithms based on proteomics, metabolomics and clinical data:**

**A systematic review**

Andrea Romano^1,2*^_,_ Tea Lanišnik Rižner^3*^, Henrica Maria Johanna Werner^1,2^_,_ Andrzej Semczuk^4^, Camille Lowy^5^, Christoph Schröder^5^, Anne Griesbeck^5^, Jerzy Adamski^6,7,3^, Dmytro Fishman^8,9^, Janina Tokarz^10,11^, BIOENDOCAR **

*^1^Department of Gynaecology, Maastricht University Medical Centre (MUMC), The Netherlands*

*^1^GROW – School for Oncology and Reproduction, Maastricht University, The Netherlands*

*^3^Institute of Biochemistry and Molecular Genetics, Faculty of Medicine, University of Ljubljana, Ljubljana, Slovenia*

*^4^Department of Gynaecology, Lublin Medical University, Poland*

*^5^Sciomics GmbH, Heidelberg, Germany*

*^6^Department of Biochemistry, Yong Loo Lin School of Medicine, National University of Singapore, Singapore*

*^7^Institute of Experimental Genetics, Helmholtz Zentrum München, German Research Center for Environmental Health, Neuherberg, Germany*

*^8^Institute of Computer Science, University of Tartu, Estonia*

*^9^Quretec Ltd., Tartu, Estonia*

*^10^Institute for Diabetes and Cancer, Helmholtz Zentrum München, German Research Center for Environmental Health Neuherberg, Germany*

*^11^German Center for Diabetes Research (DZD), Neuherberg, Germany*

*****shared first authors and corresponding authors

** BioEndoCar consortium includes partners of the Transcan2 ERA-Net project, 2018-2021.

Members of the consortium besides the authors: Špela Smrkolj, Luka Roškar, Boštjan Pirš, Department of Gynecology, University Medical Centre Ljubljana, Slovenia; Aneta Adamiak-Godlewska, Sara Wawrysiuk and Ola Kaminska, Department of Gynaecology, Lublin Medical University, Poland.

External collaborators: Iztok Takač, Jure Knez, Monika Sobočan, University Medical Centre Maribor, Slovenia; Vit Weinberger and Petra Vinklerova, Department of Gynecology and Obstetrics, University Hospital Brno, Czechia; Fabio Barra, Department of Obstetrics and Gynaecology, IRCCS Ospedale Sacro Cuore - Don Calabria, Genoa, Italy; Simone Ferrero, Academic Unit of Obstetrics and Gynaecology, IRCCS Ospedale Policlinico San Martino; Genoa, Italy.

**Supplementary Materials:**

**Supplementary Table S1: Search strategy for Pubmed and OVID Embase.**

**Supplementary Table S2:** Selected signaling questions to assess the quality of the selected manuscripts.

**Supplementary Table S3**: QUADOMICS scoring of the included proteomics studies.

**Supplementary Table S4**: QUADOMICS scoring of the included metabolomics studies

**Supplementary Table S5**: Additional quality checks and potential biases

**Supplementary Table S6**: Proteomics studies including less that 10 samples per group in the analyses.

**Supplementary Table S7**: Proteomics studies in endometrial cancer.

**Supplementary Table S8**: Metabolomics studies in endometrial cancer.

**Supplementary Table S1: Search strategy for Pubmed and OVID Embase**

| **PUBMED** |  | |
| --- | --- | --- |
|  | **Endometrial cancer AND proteomics** | |
|  | **((("Endometrial Neoplasms"[Mesh] OR "Endometrial Neoplasms"[tiab] OR "Endometrial Carcinoma*"[tiab] OR "Endometrial Cancer*"[tiab] OR "Endometrium Cancer*"[tiab] OR "Cancer of the Endometrium"[tiab] OR "Carcinoma of Endometrium"[tiab] OR "Cancer of Endometrium"[tiab]) OR ("Uterine Neoplasms"[Mesh] OR "Uterine Neoplasm*"[tiab] OR "Uterus Neoplasm*"[tiab] OR "Cancer of Uterus"[tiab] OR "Uterus Cancer*"[tiab] OR "Cancer of the Uterus"[tiab] OR "Uterine Cancer*"[tiab] OR "Uterine Carcinoma*"[tiab] ) OR ("Endometrial Neoplasms"[Mesh] OR "Endometrial Neoplasm*"[tiab] OR "Endometrial Carcinoma*"[tiab] OR "Endometrial Cancer*"[tiab] OR "Cancer of the Endometrium*"[tiab] OR "Carcinoma of Endometrium*"[tiab] OR "Endometrium Carcinoma*"[tiab] OR "Cancer of Endometrium*"[tiab] OR "Endometrium Carcinoma*"[tiab] OR "Endometrium Cancer*"[tiab])) AND** ("Proteomics"[Mesh] OR "Proteomic*"[tiab])) | |
|  | **Endometrial cancer AND metabolomics** | |
|  | **((("Endometrial Neoplasms"[Mesh] OR "Endometrial Neoplasms"[tiab] OR "Endometrial Carcinoma*"[tiab] OR "Endometrial Cancer*"[tiab] OR "Endometrium Cancer*"[tiab] OR "Cancer of the Endometrium"[tiab] OR "Carcinoma of Endometrium"[tiab] OR "Cancer of Endometrium"[tiab]) OR ("Uterine Neoplasms"[Mesh] OR "Uterine Neoplasm*"[tiab] OR "Uterus Neoplasm*"[tiab] OR "Cancer of Uterus"[tiab] OR "Uterus Cancer*"[tiab] OR "Cancer of the Uterus"[tiab] OR "Uterine Cancer*"[tiab] OR "Uterine Carcinoma*"[tiab] ) OR ("Endometrial Neoplasms"[Mesh] OR "Endometrial Neoplasm*"[tiab] OR "Endometrial Carcinoma*"[tiab] OR "Endometrial Cancer*"[tiab] OR "Cancer of the Endometrium*"[tiab] OR "Carcinoma of Endometrium*"[tiab] OR "Endometrium Carcinoma*"[tiab] OR "Cancer of Endometrium*"[tiab] OR "Endometrium Carcinoma*"[tiab] OR "Endometrium Cancer*"[tiab])) AND** ("Metabolomics"[Mesh] OR "Metabolomic*"[tiab])) | |
| **OVID EMBASE** | |  |
|  | | **Endometrial cancer AND proteomics** |
|  | | ***1:* exp endometrium tumor/**   \| ***2:*** \| ((endometrial or endometrium) adj3 (tumo?r$ or carcinoma$ or cancer$)).ti,ab. \| \| --- \| --- \|   ***3:* 1 OR 2**  ***4:* exp proteomics/**  ***5:* proteomic$.ti,ab.**  ***6:* 4 or 5**  ***7:* 3 and 6** |
|  | | **Endometrial cancer AND metabolomics** |
|  | | ***1:* exp endometrium tumor/**   \| ***2:*** \| ((endometrial or endometrium) adj3 (tumo?r$ or carcinoma$ or cancer$)).ti,ab. \| \| --- \| --- \|   ***3:* 1 OR 2**   \| ***4:* exp metabolomics/** \|  \|  \| \| --- \| --- \| --- \| \| ***5:* metabolomic$.ti,ab.** \|  \|  \|   ***6:* 4 or 5**  ***7:* 3 and 6** |

**Supplementary Table S2**: Selected signaling questions to assess the quality of the selected manuscripts.

|  | **QUADOMICS signaling questions** |
| --- | --- |
| **1** | **Was selection criteria clearly described?**  *Inclusion/exclusion criteria,*  *detailed information on sources of samples*  *(flow diagram not needed)* |
| **2** | **Was the spectrum of patients representative?**  *Target population that would need diagnostic or prognostic test.* |
| **3A** | **Was the type of sample fully described?**  *Type of sample (serum, plasma, tissue sample, etc.)*  *(for plasma: EDTA, heparin, citrate), time before centrifugation for serum!*  *centrifugation time and g (not rpm)*  *how were tissue sample obtained* |
| **3B** | **Was the collection procedure of sample fully described?**  *time of sample collection (morning, during the day, …)*  *time between blood flow and centrifugation (delay in processing)*  *time between sample acquisition and storage*  *freeze-thaw cycles*  *for tissues: time between collection and freezing* |
| **4** | **Were the procedures of biological sample collection with respect to clinical factors described with enough detail?**  *Clinical and physiological factors?*  *Age, fasting status, BMI, menstrual phase (if applicable), menopausal status* |
| **5** | **Were handling and pre-analytical procedures reported in sufficient detail and similar for the whole group?**  *If differences in procedures were reported, was their effect on the results assessed?*  *Detailed description of pre-analytical procedures: temperature of storage, procedure of metabolite/protein extraction.* |
| **6** | **Is the time between the reference standard and the index test short enough to guarantee that the target condition did not change between the two tests?**  *Samples are usually obtained before or during surgery, which is considered a reference standard.* |
| **7** | **Did the whole sample or a random selection of the sample receive verification using a reference standard of diagnosis?**  *In the case/control studies healthy controls did not undergo surgical treatment.* |
| **8** | **Was the execution of the index test described in sufficient detail to permit replication of the test?**  *Metabolomics analysis: description of the MS or NMR* ***method****,* ***control procedures****, (calibration and randomization only for MS)* |
| **9** | **Was statistical analysis of the index test described in sufficient detail?**  *Statistical methods, reproducibility assessment, normalization, transformation and cross-validation (leave-one-out, bootstrap, jackknife and permutation tests, independent training and test set)*  *Validation test performed:* ***yes****/no OR*  *Other approaches for overfitting:* ***yes****/no* |

**Supplementary Table S3**: QUADOMICS scoring of the included proteomic studies.

| **Study/QUADOMICS** | **1** | **2** | **3A^1^** | **3B^2^** | **4^3^** | **5** | **6** | **7** | **8** | **9** | **comments** |
| --- | --- | --- | --- | --- | --- | --- | --- | --- | --- | --- | --- |
| **Proteomics** |  |  |  |  |  |  |  |  |  |  |  |
| Yoshizaki 2005 tissue | Yes | No* | Yes** | NC^$^ | No^$$^ | No | Yes | NC^#^ | NC^##^ | Yes | * Controls pre-menopausal; **tissue with confirmation adjacent FFPE; $ immediatey, no mention to a max time allowed; $$ No BMI information; # unclear if all women underwent hysterectomy; ##protein extraction: protein concentration detection method not named, ProteinChip array analysis: no info if samples were pooled or not before application on the array -> one array per sample?, SELDI analysis: calibration just named as `routine calibration` without explanation |
| DeSouza 2007 tissue | No* | No** | Yes | Yes | No | Yes | Yes | Yes | NC^$^ | Yes | * Samples selected from biobank, no further info; ** Controls pre-menopausal; $ protein extraction: centrifugation details not described, labelling: no info how randomization in iTRAQ sets |
| Voisin 2011 tissue | No* | No** | No | No | No | No | No | No | NC^$^ | Yes | * No mention; 5 samples were analysed in a previous study (most probably De Souza 2007), but there is no reference; **Controls pre-menopausal; $ protein extraction: centrifugation details not described, labelling: no info how randomization in iTRAQ sets |
| Shan 2016 tissue | Yes | Yes | Yes | No | No | No | No | Yes | No | No |  |
| Ceylan 2020 tissue | Yes | Yes | Yes | yes | yes | yes | yes | yes | yes | No |  |
| Mauland 2017 tissue | yes | yes | yes | yes | yes | Yes | yes | yes | Yes* | yes | * details missing like protein extraction procedure, but authors refer to previous studies |
| Akkour 2022 tissue | yes | yes | yes | no | no | no | yes | Yes | NC* | yes | * protein extraction: no info on homogenization time, no info on how were contaminants removed (but referred to previous publications). Protein separation by 2-D electrophoresis: separated gels for identification of different spots and analysis by MS/MS: authors largely referred to previous publications |
| Kurimchak tissue | yes | yes | yes | yes | no | yes | yes | yes | yes | Yes |  |
| DeSouza 2010 tissue | No | No* | Yes | Yes | No | yes | no | NC | Yes | Yes | * proliferative endometrium as controls |
| Aboulaurd 2021 tissue | yes | yes | yes | Yes | yes | yes | yes | Yes | yes | Yes |  |
| Janakova 2021 tissue | Yes | Yes* | yes | Yes | Yes | yes | yes | yes | yes | Yes | * differences in stage and grade between the two groups |
| Zhu 2006 serum | yes | yes | No | No* | No | no | yes | No | Yes | yes | * serum was stored for max 48 h at 2°C before -80°C, no further details |
| Kikuchi 2007 serum | yes | Yes* | yes | yes | Yes | yes | yes | yes | yes | yes | * differences in mean ages between groups; (2) No validation, basic statistics |
| Zhu 2008 serum | yes | yes | no | No* | no | no | yes | no | yes | yes | * serum was stored for max 48 h at 2°C before -80°C, no further details |
| Wang 2011 serum | yes | yes | NC* | No | Yes | yes | yes | yes | yes | Yes | * no clotting time, rpm and no x g; |
| Qiu 2010 serum | yes | yes | Yes* | yes | no | yes | yes | yes | yes | Yes^$^ | * Serum was let sediment (at least 30 min, immediately aliquoted and frozen; $ algorithms not fully explained |
| Enroth 2018 plasma | yes | yes | no | no | NC* | yes | yes | yes | yes ^$^ | yes | * age only; $ not many details given but the authors refer to the Olink web page |
| Tarney 2019 serum | yes | yes | Yes | Yes* | yes | yes | yes | yes | yes | Yes | * Only study reporting the number of freeze-thaw cycles (no more than two) |
| Ura 2021 serum | yes | yes | yes | yes | yes | yes | no | yes | yes | yes |  |
| Celsi 2022 serum | yes | yes | yes | yes | yes | yes | no | yes | yes | yes |  |
| Ura 2022 serum | yes | Yes* | yes | yes | no | yes | yes | yes | Yes^$^ | yes | * mean age difference between cases and controls; $ procedures were performed at Olink® Proteomic (Dag Hammarskjölds väg 52B, SE-752 37 Uppsala, Sweden |
| Martinez-Garcia 2016 uterine aspirate | yes | NC* | yes | yes | yes | yes | yes | yes | yes | yes | * supplementary table with patient info could not be found on the journal website |
| Martinez-Garcia 2017 uterine aspirate | yes | yes | yes | yes | yes | yes | yes | yes | yes | yes |  |

1: Although indicated as ‘yes’, no study on plasma reported on EDTA, heparin, citrate and one study only reported the time of serum sedimentation.

2: Although indicated as ‘yes’, no study reported the time of sample collection, and few studies reported other info (as indicated in the table).

3: Although indicated as ‘yes’, no studies reported on the fasting status, and not always BMI was reported (as indicated in Tables).

**Supplementary Table S4**: QUADOMICS scoring of the included metabolomic studies.

| **Study/QUADOMICS** | **1** | **2** | **3A** | **3B** | **4** | **5** | **6** | **7** | **8** | **9** | **comments** |
| --- | --- | --- | --- | --- | --- | --- | --- | --- | --- | --- | --- |
| **Metabolomics** |  |  |  |  |  |  |  |  |  |  |  |
| Ihata 2014 plasma | yes | NC* | No** | NC^$^ | No*** | no | NC | no | no | no | *BD and HW; **rpm; ***no menopausal status, BMI; ^$^no information on daytime of sample aqcquisition, timing of sample processing, and freeze-thaw cycles |
| Trousil 2014 tissue | No* | no | No** | NC^$^ | No*** | yes | yes | yes | yes | NC^#^ | Normal tissue; *almost no data; **biopsy or sample after hysterectomy; ***no clinical data; **** not written-clear for tissue samples?; ^$^no information on daytime of sample acquisition and freeze-thaw cycles; ^#^ no info on data transformation and scaling |
| Jove 2016 tissue | no | No* | no | NC^$^ | No** | no | yes | yes | NC^#^ | no | *reproductive age women in control group; ** no data about age, menopausal status, BMI...; ^$^no information on daytime of sample acquisition and time between collection and storage; ^#^ no info on sample randomization and no QC samples used |
| Shao 2016 urine | yes | no | yes | NC^$^ | No** | yes | yes | no | NC^#^ | NC^##^ | *BD and HW; **no clinical data, age, BMI, menopausal status; ^$^no information on timing of sample processing; ^#^ no info on sample randomization; ^##^ no info on sample-to-sample normalization, data transformation, and scaling |
| Altadill 2017 tissue | yes | NC | yes | NC^$^ | No* | yes | yes | yes | NC^#^ | no | Benign disease; *age and BMI is missing; ^$^no information on daytime of sample acquisition; ^#^ no info on sample randomization and type of QC sample |
| Audet-Delage 2018 (Front Pharm) serum | yes | nc | No* | yes | NC** | yes | yes | No*** | no | no | Benign disease; *no data about collection and storage; **fasting status; *** HW |
| Audet-Delage 2018 (JSBMB) serum | yes | yes | No* | NC^$^ | yes | yes | yes | yes | NC^#^ | no | * no data about collection and storage; ^$^no information on timing of sample processing; ^#^ no info on sample randomization |
| Troisi 2018 serum | yes | no | No** | yes | yes | yes | NC | no | NC^#^ | NC^##^ | *BD and HW; **no data about centrifugation; ^#^ no info on sample randomization; ^##^ no info on sample-to-sample normalization |
| Shi 2018 serum | yes | No* | No** | NC^$^ | No*** | yes | NC | no | NC^#^ | NC^##^ | *HW; **time before centrifugation; *** menopausal status; ^$^no information on daytime of sample acquisition, timing of sample processing, and freeze-thaw cycles; ^#^ no info on sample randomization and QC samples; ^##^ no info on sample-to-sample normalization, data transformation, scaling |
| Knific 2018 plasma | yes | NC | yes | NC^$^ | No* | yes | yes | yes | NC^#^ | yes | Benign diseases; *fasting status; ^$^no information on daytime of sample acquisition, timing of sample processing, and freeze-thaw cycles; ^#^ no info on sample randomization |
| Bahado-Singh 2018 serum | yes | No* | No** | NC^$^ | No*** | yes | yes | no | NC^#^ | NC^##^ | *HW; **no data about serum collection;***menopausal status, fasting?; ^$^no information on daytime of sample acquisition, timing of sample processing, and freeze-thaw cycles; ^#^ no info on sample randomization and QC samples; ^##^ no info on sample-to-sample normalization and scaling |
| Cummings 2019 tissue | no | NC | No* | NC^$^ | No** | no^#^ | yes | Yes | NC^##^ | no | Normal and benign tissue; * no data about sample collection; ** no clinical data, no age for CW; ^$^no information on daytime of sample acquisition; ^#^ no storage temperature reported; ^##^ no info on sample randomization and QC samples |
| Strand 2019 plasma | yes | yes | yes | NC^$^ | No* | yes | yes | yes | no | no | *not fasting; ^$^no information on daytime of sample acquisition, timing of sample processing, and freeze-thaw cycles |
| Cheng 2019 CV fluid | yes | NC | No* | NC^$^ | No** | yes^#^ | NC | yes | yes | yes | Normal, benign diseases; *not clear what was time between collection and storage, when in the menstrual, menopausal cycle has been collected; ** premenopausal and menopausal women; ^$^no information on daytime of sample acquisition, timing of sample processing, and freeze-thaw cycles; ^#^ no sample storage reported |
| Lunde 2020 serum | Yes | Yes | No | No | No* | No | Yes | Yes | No | No | Flow diagram available; *no info about fasting status, age, BMI, menopausal status (matched on age and BMI) samples from Danish Cancer Biobank, prognosis of postoperative pain |
| Shafiee 2020 blood (serum/plasma), tissue | Yes | Yes | No | No | No* | No | Yes | Yes** | Yes | NC^$^ | *no info about fasting status, collection and storage of blood samples; **outpatient endometrial sampling for PCOS, hysterectomy or hysteroscopy for EC and C patients; ^$^ no information on missing values imputation, data transformation and scaling |
| Troisi 2020 dried blood spots | Yes | Yes* | Yes | NC^$^ | No** | Yes | Yes | No*** | NC^$$^ | NC^$$$^ | Flow diagram available; * not sufficient info about control patients; **fasting status not available; ***not clear for C in test cohort, histology for suspected EC cases in prospective cohort; ^$^ storage temperature not given; ^$$^ no information on sample randominzation and quality control; ^$$$^ no information on normalization, transformation, scaling, missing value treatment |
| Kozar 2021 serum | Yes | No* | Yes | NC^$^ | Yes** | Yes | Yes | No*** | NC^$$^ | Yes | *difference in menopausal status, age, fasting, no alcohol, smoking, medication (time frame not clear); *** ultrasound for C patients; ^$^ time of day not clear; ^$$^ no information on sample randomization and MS parameters |
| Njoku 2021 plasma | Yes | Yes* | NC^$^ | No | Yes** | Yes | Yes | No*** | NC^$$^ | Yes | *prognosis of EC in obese population; **overnight fast; *** endometrial biopsy for C patients, biopsy or hysterectomy specimen for EC patients; $ centrifugal force not given; $$ no info on sample randomization and QC samples |
| Kliemann 2021 plasma and serum | Yes* | Yes** | NC | No | Yes | Yes | NC*** | No# | NC^$^ | Yes | *no flow diagram; ** metabolic signatures typical for obesity and EC; *** time between reference and index test is not known; # HW; ^$^ no information on sample randomization and QC samples |
| Dossus 2021 plasma | Yes* | No** | NC | No^$^ | Yes | Yes | NC*** | No# | Yes | Yes | *no flow diagram; ** no info about the control group, probably HW; *** time is known but is long 8.3 (4.5); # HW?; ^$^ timing of sample collection and storage temperature unclear |
|  |  |  |  |  |  |  |  |  |  |  |  |
| Skorupa 2021 tissue | Yes* | Yes** | Yes | NC^$^ | Yes*** | Yes | Yes | Yes | NC^$$^ | NC^$$$^ | *no flow diagram; ** patients with different grades of EC; *** fasting status not available (not needed for tissue metabolomics); ^$^ time of day of sample collection not given; ^$$^ no information on metabolite identification; ^$$$^ no information on missing value treatment |
| Gu 2021 serum | Yes* | Yes** | No | Yes | No*** | Yes | Yes# | Yes## | NC^$^ | NC^$$^ | *no flow diagram;** patients with different stages of EC; ***no age, BMI…; # samples collected on the morning of surgery, all patients underwent hysterectomy; ^$^ no information on sample randomization, use of QC samples and metabolite identification; ^$$^ no information on missing value imputation, transformation and scaling |
| Yan et al. 2022, serum | Yes* | Yes** | NC^$^ | NC^$$^ | NO*** | NC^$^ | NC# | NC## | NC^$$$^ | NC^$$$$^ | *no flow diagram; **patients with hyperplasia, polyps; *** no info about fasting status and BMI; #no info about the time of blood collection; ## not clear what kind of diagnosis was used for EP and EH; ^$^ centrifugal force not given in x g; ^$$^ time of day of sample collection not given; ^$$$^ no information on sample randomization; ^$$$$^ no information on data transformation and scaling |
| Schuhn 2022 serum | Yes* | Yes** | Yes | NC^$^ | NO*** | Yes | NC# | No## | NC^$$^ | No^$$$^ | *no flow diagram; **other cancers (breast) benign endometrial diseases, HW; *** no fasting and menopausal status; #no info about the time of blood collection; ## breast cancer, control women and healthy women did not have the same diagnostic test; ^$^ time of day of sample collection not given; ^$$^ no information on sample randomization and QC samples; ^$$$^ no information on sample-to-sample normalization, transformation, scaling, model calculation, crossvalidation and approaches for overfitting |
| Düz 2022 tissue | Yes* | Yes** | Yes | No^$^ | NO*** | No^$$^ | Yes# | Yes## | Yes | NC^$$$^ | *no flow diagram; ** benign endometrial pathologies versus cancer; *** no info about BMI, grade, stage of cancer, menopausal status (however, authors state the samples were matched for age, BMI and menopausal status); # tissue samples were obtained during hysterectomy; ## all patients underwent hysterectomy; ^$^ timing unclear; ^$$^ buffer compostion and centrifugation details missing; ^$$$^ no information on missing value treatment, transformation; permutation test number too low |
| Yie 2022 tissue, urine, brushing samples | Yes* | NC** | Yes | NC^$^ | NO*** | NC^$$^ | Yes# | NC## | Yes | NC^$$$^ | *no flow diagram; ** control group included also patients with cervix diseases, no info about number of different pathologies; *** BMI, age menopausal status availabe, but there was no data about menstrual phase for premenopausal women, fasting not available, but not relevant; # tissue samples were obtained during hysterectomy and urine one day before surgery, not clear for intrauterine brushing; ## not clear whether hysterectomy with histology has been performed for all patients; ^$^ time of day and time between collection and storage unclear; ^$$^ centrifugal forces not given in x g; ^$$$^ no information on missing value treatment |
| Gatius 2022 tissue | Yes* | Yes** | No# | No# | NO*** | Yes | NC# | Yes## | NC^$^ | No | *no flow diagram; **separation between endometrioid and serous EC; *** no data about BMI and menopausal status; # no info provided on samples from biobank; ## no info; ## it is logical, but is not described; ^$^ no information on sample randomization and QC samples |
| Breeur 2022 blood | Yes* | Yes** | Yes | NC^$^ | No*** | NC^$$^ | Yes# | No## | NC^$$$^ | Yes | *no flow diagram; **for epidemiological study, diagnostic biomarkers specific fof EC in whole population; *** no data for menopausal status; # info provided, relatively long 8.4 years after blood collection; ## only EC patients; ^$^ no more information than »standardized protocols«; ^$$^ sample preparation not described; ^$$$^ no information on sample randomization, QC samples and measurements |

**Supplementary Table S5:** Additional quality checks and potential biases

| **Study** | **Country** | **Funding** | **COI** | **Open science** |
| --- | --- | --- | --- | --- |
| (Yoshizaki, Enomoto et al. 2005) | Japan | No mention | No mention | No mention |
| (DeSouza, Grigull et al. 2007) | Canada | National Cancer Institute of Canada;  Ontario Genomics Institute and Genome Canada | No mention | No mention |
| (Voisin, Krakovska et al. 2011) | Canada | Support by Canadian Cancer Society Research Institute and MITACS Accelerate Fellowship, Ontario | No mention | No mention |
| (Shan, Zhou et al. 2016) | China | No mention | No mention | No mention |
| (Ceylan, Akpinar et al. 2020) | Turkey | No mention | No mention | No mention |
| (Mauland, Ju et al. 2017) | Norway | University of Bergen;  Western Norwegian Regional Health Authority; Research Council of Norway;  Norwegian Cancer Society. | None | No mention |
| (Akkour, Alanazi et al. 2022) | Saudi Arabia | Funding: Dallah Hospital Group in Riyadh | None | No mention |
| (Kurimchak, Kumar et al. 2068) |  | National Institutes of Health CORE Grant (Fox Chase Cancer Center)  R01  National Institutes of Health  Donations from Peggy’s Pathway for Women’s Cancer Care Cancer Kinome Initiative (CKI) at FCCC  Donation from Don Morel | None | No mention |
| (DeSouza, Krakovska et al. 2010) | Canada | Canadian Cancer Society Research Institute  Applied Biosystems/MDS Analytical Technologies | None  Authors acknowledge company support | No mention |
| (Aboulouard, Wisztorski et al. 2021) | France, Lebanon | Ministere de L’Education Nationale, de L’Enseignement Superieur et de la Recherche  SIRIC ONCOLille  INCa-DGOS-INSERM | None | No mention |
| (Janacova, Faktor et al. 2617) | Switzerland, Czech Republic  Ireland | Czech Science Foundation | None | Deposited data ProteomeXchange Consortium / PRIDE partner repository (PXD017217) |
| (Zhu, Zhang et al. 2006) | China | No mention | No mention | No mention |
| (Kikuchi, Honda et al. 2007) | Japan | Foundation for the Promotion of Cancer Research (Tokyo, Japan)  Ministry of Health, Labor and Welfare of Japan  Ministry of Education, Culture, Sports, Science and Technology of Japan  National Institute of Biomedical Innovation of Japan  Naito Foundation | No mention | No mention |
| (Zhu, Zhang et al. 2008) | China | Research Grants from Peking University | No mention | No mention |
| (Qiu, Gao et al. 2010) | China, USA | Ministry of Science and Technology of China | No mention | No mention |
| (Wang, Cao et al. 2011) | China | Shanghai Leading Academic Discipline Project  Shanghai fundamental research emphasis project | None | No mention |
| (Enroth, Berggrund et al. 2018) | Sweden | The Swedish Cancer Foundation Vinnova (SWELIFE)  The Foundation for Strategic Research (SSF)  Assar Gabrielsson Foundation | None | Authors declare that data would be available  upon publication |
| (Tarney, Wang et al. 2019) | USA | Uniformed Services University of the Health Sciences from the Defense Health Program | None | No mention |
| (Ura, Biffi et al. 2021) | Italy | Italian Health Ministry | None | Data available on request (for ethical reasons) |
| (Celsi, Monasta et al. 2022) | Italy | Italian Health Ministry | None | Data available on request |
| (Ura, Capaci et al. 2022) | Italy | Italian Health Ministry | None | Data available on request (for ethical reasons) |
| (Martinez-Garcia, Lesur et al. 2016) | Spain, USA, Luxemburg | Spanish Ministry of Health  Spanish Ministry of Economy and Competitivity  Spanish Ministry of Education, Culture and Sport  Fondo Europeo de Desarrollo Regional - FEDER  Grupos Estables de Investigacion 2011 – AECC  Fundació La Marató TV3  CIRIT Generalitat de Catalunya  European Commission, 7th Framework Programme, IRSES  "Fonds National de la Recherche du Luxembourg" (FNR)  AFR grant | None | No mention |
| (Martinez-Garcia, Lesur et al. 2017) | Spain, USA, Luxemburg | Spanish Ministry of Health  Spanish Ministry of Economy and Competitivity  Fondo Europeo de Desarrollo Regional  Grupos Estables de Investigacion 2011-AECC  Fundació La Marató TV3  CIRIT Generalitat de Catalunya Fundación DEXEUS Salud de la Mujer  Spanish Ministry of Economy and Competitiveness  PERIS grant (Generalitat de Catalunya)  Fonds National de la Recherche du Luxembourg" (FNR)  AFR grant | None |  |
| (Ihata, Miyagi et al. 2014) | Japan | Not mentioned | Several authors are employed by Ajinomoto Co. | No |
| (Trousil, Lee et al. 2014) | UK | National | No | No |
| (Jove, Gatius et al. 2016) | Spain | National, Eu grants | No | no |
| (Shao, Wang et al. 2016) | China | National | no | no |
| (Altadill, Dowdy et al. 2017) | Spain | National, Eu grants | no | yes |
| (Audet-Delage, Villeneuve et al. 2018) | Canada | national | Not mentioned | yes |
| (Audet-Delage, Grégoire et al. 2018) | Canada | national | no | no |
| (Troisi, Sarno et al. 2018) | Italy | Theoreo Srl spin-off University of Salerno | Applied for a patent | no |
| Shi et al. 2018 | China | National | no | no |
| (Knific, Vouk et al. 2018) | Slovenia | National | no | no |
| (Bahado-Singh, Lugade et al. 2017) | USA | Not mentioned | no | no |
| (Cummings, Massey et al. 2019) | UK | National | Not mentioned | No |
| (Strand, Tangen et al. 2019) | Norway  Netherlands | National | No | Yes |
| (Cheng, Chen et al. 2019) | Taiwan | national | no | No |
| (Lunde, Nguyen et al. 2020) | Denmark | National funding | no | yes |
| (Shafiee, Ortori et al. 2020) | UK, Malaysia, | National funding | no | yes |
| (Troisi, Raffone et al. 2020) | Italy | National funding, | Yes  CEO Theoreo, patent owners,  Hosmotic, Theoreo | yes |
| (Kozar, Kruusmaa et al. 2021) | Slovenia | Universal DX | Yes  Employees of  Universal DX |  |
| (Njoku, Campbell et al. 2021) | UK | Natinal Funding | No | Yes |
| (Kliemann, Viallon et al. 2021) | France | European and national funding | No | Yes |
| (Dossus, Kouloura et al. 2021) | UK | National funding | no | no |
| (Skorupa, Ponski et al. 2021) | Poland | national funding | No | Yes |
| (Gu, Chen et al. 2021) | China | National funding | Not mentioned | no |
| (Yan, Zhao et al. 2022) | China | National funding | no | no |
| (Schuhn, Tobar et al. 2022) | Germany | National funding | Patent applicaton | yes |
| (Arda Düz, Mumcu et al. 2022) | Turkey | National funding | No | No |
| (Yi, Xie et al. 2022) | China | National funding | Yes; employees of Shanghai Omicsolution ltd. | yes |
| (Gatius, Jove et al. 2022) | Spain | National funding | no | Yes |
| (Breeur, Ferrari et al. 2022) | France | National funding | no | yes |

**Supplementary Table S6:** Proteomic studies with less than 10 samples

| **Study**  **Aim** | **Samples** | **Study design**  **Verification** | **Method** | **Control group** | **Case group** | **Note/findings** |
| --- | --- | --- | --- | --- | --- | --- |
| (DeSouza, Diehl et al. 2005)  Diagnostic Biomarkers | Frozen tissue | Case - Control | iTRAQ / cICAT | N=3  **iTRAQ** 1 proliferative endometrium  1 secretory endometrium  **cICAT** 1 proliferative endometrium | N=5  **iTRAQ** 2 ECs  **cICAT** 3 ECs | histology confired on mirror face of the tissue block |
| (Li, Huang et al. 2008, Li, Zhao et al. 2008, Li, Min et al. 2010)*  Diagnostic & Prognsotic Biomarkers | Frozen tissue | Case – Control  Proteomics on test set + HIC verification | MALDI-Q-TOF MS | N=0 for proteomics  Validation by HIC on FFPE: 29 endometrial intraepithelial neoplasia, 39 premonopausal endometria | N=8 for proteomics  EC + adjacent tissue 45.6±3.4y (41-50y) Stage 1: 7  Stage 2: 1  Validation: HIC on FFPE (n=84) | Candidate potential biomarkers identified, including epidermal fatty acid-binding protein, calcyphosine, and cyclophilin A |
| (Attarha, Andersson et al. 2013)  Biomarkers predictive of individualised tomor features | Frozen tissue | Cases only  Proteomics on test set + HIC verification (TMA) | DIGE-MALDI-TOF | N=0 for proteomics  Validation by HIC 168 ECs and controls (not further specified) | N=3 for proteomics  EC + adjecent tissue  Validation by HIC 168 ECs and controls (not further specified) | PKN1 and MST1 associated with aggressive cancer features |
| (Lintel, Luebker et al. 2018)  Diagnostic Biomarkers (leiomyoma vs leiomyosarcoma) | FFPE Tissue | Case – Control  Proteomics on test set + HIC verification (TMA) | LC-ESI-MS/MS | N=5 for proteomics  Leiomyoma  Validation by HIC on TMA: 15 Leiomyoma (including those used for proteomics) | N=5 for proteomics  Leiomyosarcoma  Validation by HIC on TMA: 8 Leiomyosarcoma (including those used for proteomics) | Samples from FFPE archive  592 proteins quantified, 10 DEP  MVP (Major vault protein)  Sensitivity: 50%  Specificity: 100%  COMT (Catechol O-methyltransferase)  Sensitivity: 38%  Specificity: 88% |
| (Liu, Hong et al. 2020)  Diagnostic Biomarkers | Tissue (probably fresh froze, not mentioned) | Case – Control  Proteomics on test set + HIC verification | 2D LC-MS/MS quantitative proteomics | N=5 for proteomics  Validation by HIC: 30 normal endometria (including those used for proteomics) | N=5 for proteomics  Validation by HIC: 30 ECs (including those used for proteomics) | 2521 proteins quantified, 619 DEP  PI3K/AKT/mTOR pathway-related molecules (including PI3K, mTOR, ERK, SPP1, ANGPT2) associated with cancer |
| (Uyar, Huang et al. 2021)  Diagnostic biomarkers by comparing the proteome before and after surgery in patients | serum | Case – control  Confirmation by western blotting, in vitro and in the TCGA dataset | 1D PAGE + LC-MS/MS | N=4  benign conditions undergoing hsyterectomy | N=8  EC, prior and after surgery | several DEP indeitified  FAM83D confirmed by western blotting, in vitro and in the TCGA dataset (association with grade) |
| (Mu, Lim et al. 2012)  Diagnostic Biomarkers | Urine | Case - Control | DIGE/MALDI TOF MS/MS | N=11  Age matched | N=7  Newly diagnosed  Stages IB-IIIB | DEP: 🡩 alpha-1 acid glycoprotein (AAG), zinc alpha-2 glycoprotein (ZAG)  🡫CD59 |
| (Mu, Lim et al. 2016)  Test set  Diagnostic Biomarkers (glycopeptides) | 2016 | Urine  Morning midstream urine (50mL) | SELDI-TOF | N=4  Heatly volunteers | N=4  ECs | Potential candicate biomarkers (m/z peaks) identified |
| (Ura, Monasta et al. 2017)  Diagnostic & Prognostic Biomarkers | uterine aspirate | Case – Control  Validation by western blotting | DIGE-MS | N=6 | N=10 | 25 DEP  Including ABRACL, PGAM2, FGB, ANXA3, validated by western blotting |
| (Alonso-Alconada, Santacana et al. 2015)  Prognostic Biomarkers | Frozen tissue | Cases only  Discovery  IHC verification  Blood verification | DIGE-MS | N=0 for protoemics  IHC: 38 Normal Endometria  Serum: 27 age matched controls | N=12 for proteomics  Primary / matched recrrent EC  IHC: cohort 1: 140 EC (115 primary and 25 postradioation recurrecne); Cohort 2: 131 primary EC (93 EEC of which 50 reccurring and 43 non-recurrent; 38 NE  serum: 34 EC (from Grade 3 Stage IB to tage IV / recurrences); 27 age matched controls | macroscopically dissected endometrial primary lesions  ANXA2 by IHC (threshold od 190)  Sensitity: 76% (62–87% 95% CI) Specificity: 67% (51–81% 95% CI) AUC: 74% (ROC analysis) |
| (Huang, Hao et al. 2021)  Diagnostic biomarkers | Frozen tiessue | Cases only  Verification by IHC (on controls as well) | label‐free quantification (LFQ) (LC‐MS/MS) | N=0 for proteomics  FFPE cohort (retrospective): 30 controls  50.9y (46‐58y) | N=3 for proteomics (prospective)  EC + adjacent normal tissue  FFPE cohort (retrospective):  75 EC  51.7y (42‐76y)  Stage I-II: 54  Stage III-IV: 21 | 3245 proteins identified, 925 DEP  🡩: IFIT3, pPARP9, SLC34A2, CYB5R1, PTPN1 🡫: DPT, SLPI |
| (Mittal, Klingler-Hoffmann et al. 2016, Mittal, Klingler-Hoffmann et al. 2017)*  Biomarkers for patient startification (prediction LN metastasis) | FFPE tissue | Cases only  Verification by IHC | MALDI-MSI on TMA, LC-ESI-MS/MS | - | N=10 for proteomics  5 with LN metastasis, 5 without  Validation by HIC (including those used for proteomics):  Without LN metstasis: 27 With LN metastases: 16 Stage I: 27  Stage III: 16  Grade 1: 20  Grade 2: 14  Grade 3: 9 | histologyic confirmation on serial slides  based on 19 peaks m/z, 88% of the ptients were correctly cliassified.  Plectin, alpha-Actin-2, validated with LC-MS/MS DIA and immunohistochemistry |
| (Ura, Monasta et al. 2017)  Diagnostic & Prognostic Biomarkers | uterine aspirate | Case - Control | 2D-DIGE-MS | N=6 | N=10 | Candidate biomarkers were identified |

* It seems that authors used the same study cohort/data, but this is not clearly stated in the papers.

**Supplementary Table S7**: Proteomics studies in endometrial cancer.

| **Study**  **Aim** | **samples** | **Study design**  **Verification ^(1)(2)^** | **Method** | **Control group ^(2)^** | **Case group ^(2)^** | **Findings ^(3)^** |
| --- | --- | --- | --- | --- | --- | --- |
| **Blood (serum, plasma), urine and other fluids** | | | | | | |
| Zhu, 2006 (Zhu, Zhang et al. 2006)  Diagnostic Biomarkers | Serum (pre-surgical) | Case - Control | SELDI-TOF-MS | N=30  age-matched volunteers | N=40 EC  Stage I: 33; Stage II: 2  Stage III: 4; Stage IV: 1  57 y (34–78) | SN: 92.5%; SP: 100%  Based on 13 biomarkers, with total coincidence of 95.7% |
| Kikuchi, 2007 (Kikuchi, Honda et al. 2007)  Diagnostic Biomarkers | Serum | Case - Control | MALDI-TOF-MS | N=107  Metroptosis patients (n=16), 65.8±9.8y  Myoma uteri (n = 74), 48.8±4.1y  healthy volunteers (n = 17) 36.7±12.4y | N=92  Stage 0: 6; Stage I: 63  Stage II: 13; Stage IV: 2  59.4±10.5y | 507 protein peaks identified, and 3 m/z peaks distinguished ECs/controls  cut-off values of mean of controls ±2SD:  m/z 4769: SN=42.4%; SP=100%  m/z 6254: SN=38.0%; SP=97.0%  m/z 11792: SN=47.8%; SP: 97.0%  Three peaks together:  SN: 65.2% (60/92)  SP: 93.9% (31/33) |
| Zhu, 2008 (Zhu, Zhang et al. 2008)  Diagnostic Biomarkers | Serum | Case - Control |  | N=40  Discovery cohort (n=30)  Age-matched volunteers  Same as (same as in Zhu 2006)** not clearly stated by the authors  Validation cohort (n=10) | N=60  Discovery cohort (n=40). Same as in Zhu 2006)** not clearly stated by the authors  Validation cohort (n=20)  Stage I: 18; Stage II: 2 | Discovery cohort: SP: 100%; SN: 92.5%  Validation cohort SP: 60%; SN: 75%  Based on 4 protein peaks |
| Qiu, 2010 (Qiu, Gao et al. 2010)  Diagnostic Biomarkers | Serum  (pre-operative) | Case - Control | Magnetic bead separation, MALDI-TOF-MS | N=30  47y | N=30  EC, 50y | Algorithm ClinProTool: SN: 97.62%; SP: 100%  Based on 4 protein m/z peaks (2902.49, 5068.89, 6052.9, 7010.58)  Algorithm SNN SN: 92.02%; SP: 100%  Based on 10 protein peaks  Algorithm QC SN: 92.86%; SP: 93.75%  Based on 2 protein m/z peaks (1012.6, 6093.12) |
| Wang, 2011 (Wang, Cao et al. 2011)  Diagnostic Biomarkers | Serum | Cases only  (pre-malignant to malignant conditions) | iTRAQ labelling, 2D LC−MS/MS | - | N=16  Simple EH: 6; 46y (43-52y)  Complex EH: 4; 40y (28-46y)  Atypia EH: 4; 33y (29-40y) Stage I EC: 6; 53y (44-62y) | potential markers: SERPINA3; SERPINC1; APOA4; APOC2; APOE; HP; HRG; IGFBP4; ITIH4; ORM1; SAA1; SAA2 |
| Enroth, 2018 (Enroth, Berggrund et al. 2018)  Diagnostic Biomarkers | Plasma | Case - Control | Proximity Extension Assays (PEA) | N=223  Discovery cohort (n=105)  Benign tumours; 60y (16-88y)  Validation cohort (n=118)  Benign tumours (n=61); 76y (22-88y)  Healthy controls (n=57); 57y (28-86y) | N=302  Discovery cohort (n=74)  Stage I: 50; Stage II: 8  Stage III: 10; Stage IV: 5  60y (29-86y)  Validation cohort (n=228)  Stage I: 109; Stage II: 13  Stage III: 122; Stage IV: 11  Unknown: 73  68y (29-90y) | Discovery  EC vs Benign conditions SN: 70%; SP: 67%; AUC: 0.72  Based on 16 DEP  Validation EC vs Benign conditions SN: 77%; SP: 79%; AUC:0.83 EC vs healthy controls  SN: 69%; SP: 78%; AUC: 0.71  Based on 9 DEP (PRSS8, MK, WFDC2 (HE4), ADM, MMP-7, ST2, VEGF-A, IL-6, HGF) |
| Tarney, 2019 (Tarney, Wang et al. 2019)  Diagnostic Biomarkers | Serum  (prediagnostic) | Nested case-control study from larger trial | tandem-mass tag (TMT)  isobaric labelling, LC-MS/MS | N=112  matched 1:1 on age (mean, 62.1y), race, study site, year of blood draw, and year of randomization | N=112  Incident cases, 62.3y  EEC: 97  Garde 1: 52; Grade: 32  Grade 3: 5; Unknown: 8  Mixed or type 1: 15 | 6 proteins (CFB, TF, CAT, PSMB4, B2M, PCDH18)  AUC: 0.80 - 95% CI: 0.72-0.88  **Cut-off of 0.5** SN: 45.2%; SP: 96.4% NPV: 86.4%; PPV: 77.8% |
| Ura, 2021 (Ura, Biffi et al. 2021)  Diagnostic biomarkers | Serum | Case - Control | 2D-DIGE, LC-MS/MS | N=15  non-cancer patients | N=15  45y (36-48y)  Type 1 EC | 🡩 6 proteins (CLU, SERPINC1, ITIH4, C1R, APOC3, DSC1)  🡫 6 proteins (APCS, C9, APOA1, ALB, ITIH2, APOA4, ITIH2, CFHR1, ITIH2, ACTB) |
| Celsi, 2022 (Celsi, Monasta et al. 2022)  Diagnostic Biomarkers | Serum | Case - Control | 2D-DIGE, LC-MS/MS  Proteomics on 10 ECs + 10 controls (serum)  Validation by WB in 30 ECs + 30 controls | N=60  non-cancer controls  42y (32-77y)  Uterine leiomyomas (hysterectomy tissue available); 30  Serum only: 30 | N=44  45y (33- 56y) | 🡩 7 proteins (fold change ≥1.5; (APOC3, APOC2, APOE, SERPINC1, C1R, SERPINA1, A2M)  🡫 17 proteins (fold change ≤0.6; APOA1, APOA1, APCS, APOE, CLU, CD5L, CFHR1, VTN, C9, C8A, ALB, C4BPA, IGHM, ITIH2, C1R, SERPINA1, FLG2, SBSN, APOA4, CPS1)  Further focus on SBSN |
| Ura, 2022 (Ura, Capaci et al. 2022)  Diagnostic Biomarkers | Serum | Case - Control | Proximity Extension Assays (PEA)  Panel: Immuno-oncology  Panel: Target 96 Oncology III | N=44  67y (22-77y)  non-cancer controls | N=44  67y (44-81y)  Type 1 ECs | **DEP**: Gal-9, Gal-1, MMP7, FASLG, CGB3, HSPB6, CDHR2, NCS1 DCTPP1 LMLN ALPP SCLY CD300E rfng LACTB2 CCT5 GFER VPS37A VAT1 PSMD9 KLK4 CPVL FLT3 HMBS UBAC1 HLA-E AFP COL9A1, CDHR2, NCS1, MLN, FLT3, COL9A1  **Model 1 -** Immuno-oncology panel  Pseudo R-squared: 0.605  AUC: 0.954% (95% CI 0.91–0.993)  SN: 97.67%; SP: 74.42% based on 4 markers (Gal-9, Gal-1, MMP7, FASLG)  **Model 2 -** Target 96 Oncology III  Pseudo R-squared: 0.436  AUC: 0.889% (0.821–0.956)  SN: 95.45%; SP: 69.77% based on 5 markers (CDHR2 NCS1, MLN, FLT3, COL9A1)  **Model 3 -** Models 1 & 2 + COL9A1  Pseudo R-squared: 0.691  AUC: 0.969% (0.939–0.999)  SN: 97.67%; SP: 83.72% |
| Martinez-Garcia, 2016 (Martinez-Garcia, Lesur et al. 2016)  Diagnostic Biomarkers | uterine aspirate | Case - Control | LC-MS/MS, LC-PRM (parallel reaction monitoring, 52 proteins measured) | N=18  Non-cancer controls  Postmenopausal subjects  >50y | N=20  Postmenopausal subjects  >50y | 🡩 (in EC) 26 proteins (PERM, CADH1, SPIT1, ENOA, MMP9, LDHA, CASP3, KPYM, PRDX1, OSTP, PDIA1, NAMPT, MIF, CTNB1, K2C8, ANXA2, CAPG, FABP5, MUC1, CAYP1, XPO2, NGAL, SG2A1, ANXA1, HSPB1, PIGR)  ROC: 0.75 - 0.97  AUC: >0.9 (for 10 markers)  As single markers  SN: >80%; SP: 95%  4 proteins as single markers (PERM, CADH1, SPIT1, OSTP isoform A) |
| Martinez-Garcia, 2017 (Martinez-Garcia, Lesur et al. 2017)  Diagnostic Biomarkers Prognostic Biomarkers | uterine aspirate | Case - Control | LC-PRM (52 proteins measured)  Prospective training + Leave-one-out validation  Independent cohort (as in Martinez-Garcia 2016) | N= 47  Non-cancer controls (women with suspicion of EC based on endometrial thickness )  53y (30-80y) | N=69  Stage I: 43; Stage II: 12  Stage II: 10; Stage IV: 4  EEC: 49; 67y (37-87y)  Grade 1: 5; Grade 2: 33  Grade 3: 10  SEC: 20; 73y (51-93y) | 🡩 28 proteins (EC vs controls): LDHA, KPYM, aKPYM, MMP9, NAMPT, SPIT1, CADH1 , ENOA, PERM, CAPG, CH10, CTNB1, K2C8, CLIC1, PDIA1, PRDX1, CD44, MIF, FABP5, XPO2, TPIS, CASP3, GSTP1, ANXA1, NGAL, ANXA2, GTR1, OSTP: isoform A, B, D, MUC1  AUC of 5 best individual biomarkers:  LDHA: 0.91 (95% CI, 0.856–0.957)  KPYM isoform M1-M2: 0.90 (95% CI, 0.841–0.953)  MMP9: 0.89 (95% CI, 0.827– 0.950)  NAMPT: 0.88 (95% CI, 0.824–0.942)  SPIT1: 0.88 (95% CI, 0.814–0.948)  Stage Ia (n=30) vs controls (n=47) AUC >0.84 based on 5 individual biomarkers (LDHA, KPYM, MMP9, NAMPT, SPIT1)  EH (n=9) vs controls (n=38)  AUC>0.85 based on 16 proteins  **Diagnostic model (EC vs controls)** MMP9, KPYM  **Diagnostic model (EC vs controls)** Training cohort SN: 94.2% (88.4-98.6); SP: 87.2% (76.6-95.7) AUC: 0.96 (95% CI, 0.94–0.99) Leave-one-out validation SN: 89.9%; SP: 85.1% Independent cohort validation SN: 100%; SP: 83.3% (66.7-100) AUC: 0.96 (95% CI, 0.89–100)  Based on MMP9, KPYM  **Prognostic model (EEC vs SEC)** Training cohort SN: 95% (95% CI, 85–100) SP: 95.9% (95% CI, 89.8–100)  AUC: 0.99 (95% CI, 0.90–1.0) Leave-one-out validation SN: 95%; SP: 89.8%  Based on CTNB1, XPO2, CAPG |
| **Studies on Frozen Tissue** | | | | | | |
| Yoshizaki, 2005 (Yoshizaki, Enomoto et al. 2005)  Diagnostic Biomarkers | Frozen tissue | Case - Control | SELDI-TOF-MS | N=20  premenopausal (adenomyosis, myoma, cervical cancer, benign ovarian tumours) | N=19  All EEC, stage I-III  grade 1: n=4; grade 2: n=8  grade 3: n=7 | one protein consistently up-regulated  one consistently down-regulated |
| DeSouza, 2007 (DeSouza, Grigull et al. 2007)  Diagnostic Biomarkers | Frozen tissue | Case - Control | iTRAQ labelling, SCX separation, MD LC-MS/MS | N=20  proliferative endometria: n=10 secretory endometria: n=10 | N=20  Type 1 EC: n=10  Type 2 EC: n=10 | Model based on: AAT, PK, CPN10  SN: 95%; SP: 95% AUC 0.96; PPV 0.95 |
| Voisin, 2011 (Voisin, Krakovska et al. 2011)  Diagnostic, Prognostic, Therapeutic Biomarkers | Frozen tissue | Case - Control | iTRAQ labelling, drill-down LC-MS/MS | N=10  proliferative endometria | N=10  Type 1 EC (it is stated, 5 were analysed in a previous study, but no clear reference) | PPV: 1.0%; AUC: 1.0%  Based on: CALU, KRT8, CAP-G |
| Shan, 2016 (Shan, Zhou et al. 2016)  Diagnostic Biomarkers | Frozen tissue | Cases only  Verification: qRT-PCR; WB;  functional studies on cell lines (for HSPA8 only) | iTRAQ-MS | - | N=10  All EC stage I; adjacent non-cancerous tissue | **DEP**: CCT7, HSPA8, PCBP2, LONP1, PFN1, and EEF2 |
| Ceylan, 2020 (Ceylan, Akpinar et al. 2020)  Diagnostic Biomarkers | Frozen tissue | Case - Control | 2D-DIGE, MALDI TOF/TOF-MS | N=13  Benign disease with abnormal bleeding  pre-menopausal: n=7 (46.5 y) atrophic endometria: (46-64 y) complex/atypia EH: n=5 (51-63 y) | N=18  Stage IA: n=5; Stage IB: n=5  Stage II: n=3; Stage III: n=5  Age 40-78 y | EC vs EH: 19 DEP  EC stage IA vs controls:  🡩 GRP78, GSTP1, ACTG, PDIA3, ENOA  🡫 ALBU  EC stage IB vs controls:  🡩 GSTP1, ACTB, ACTG, K2C8, ANXA1, ENOA  🡫 TRFE  EC stage II vs controls:  🡩 GSTP1, PDIA3  EC stage III vs controls:  🡩GSTP1, ACTB, K2C8, PDIA3, TRFE, ENOA  EH vs controls:  🡩 HSPB1, EF-Tu, IDHC |
| Mauland, 2017 (Mauland, Ju et al. 2017)  Prognostic Biomarker associated with obesity | Frozen tissue | Cases only  Verification:  mRNA  IHC | RPPA (163 proteins measured) | - | N=518  EEC Training Norway cohort (n=272); Stage I-IV; Grade 1-3; pre/postmenopausal; 65.2±11.5 y; BMI 29.4±6.9  Validation cohort 1: Norway Test Cohort (n=68); Stage I-III; Grade 1-3; pre/postmenopausal; 66.9±10.9 y; BMI=29.1±6.0  Validation cohort 2:  MDACC, Houston, TX (n=178); Stage I-IV; Grade 1-3; pre/postmenopausal;  60.5±12.9 y; BMI 36.3±11.2 | Low PI3K-activation in non-obese patients with FIGO stage 1 and ERα positive tumours: reduced disease specific survival |
| Akkour, 2022 (Akkour, Alanazi et al. 2022)  Diagnostic Biomarkers | Frozen tissue | Case - Control | 2D-DIGE, MALDI-MS/MS | N=12  undergoing hysterectomy  Age-matched | N=24  12 EC; 12 EH; 46–75y | **DEP** potentially associated with progression from EH to EC  DES, PPIA, ZNF844, ALDOA, ENO1, KRT10 |
| Kurimchak, 2020 (Kurimchak, Kumar et al. 2020)  Prognostic Biomarkers | Frozen tissue | Cases only  Verification:  CPTAC & TCGA  IHC (n=57; SEC: 39; EEC: 18; Normal n=12)  functional validation *in vitro* | LFQ  super-SILAC labelling,  MIB-MS | - | N=20  primary EC n=20  (EEC=17; SEC: 3)  normal endometrial tissues (adjacent to tumour) n=16 | SRPK1 associated with poor survival |
| **FFPE Tissue** |  |  |  |  |  |  |
| DeSouza, 2010 (DeSouza, Krakovska et al. 2010)  Discovery  Diagnostic Biomarkers | FFPE tissue | Case - Control | mTRAQ labelling,  LC–multiple reaction monitoring (MRM)-MS, Targeted proteomics (17 proteins) | N=15  Proliferative endometria | N=10 | Feasibility of using FFPE samples |
| Aboulouard, 2021 (Aboulouard, Wisztorski et al. 2021)  Prognostic Biomarkers  (Lymph-node disease) | FFPE tissue | Case - Control | LC−MS/MS | N=9  Healthy Endometrium: 6  Normal SLN: 3 | N=15  59-74 y; BMI 26.7-41.3; Caucasian  32 samples (15 used for proteomics):  Grade I EEC: 4; Grade II EEC: 8; Grade III EEC: 4;  Grade I SNL: 4; Grade II SLN: 8 Grade III SNL: 4 | **DEP**: 1005  pathways altered in cancer  **DEP EC vs SLN**: PRSS3, PTX3, ASS1, ALDH2, ANXA1 (verified by IHC) |
| Janacova, 2020 (Janacova, Faktor et al. 2020)  Prognostic Biomarkers in the context of tamoxifen users | FFPE tissue | Cases only  EC with / without previous exposure to Tamoxifen | LC−MS/MS in SWATH-MS mode DIA | - | N=36  Tamoxifen user: 15; 67.2±8.8 y  Tamoxifen naïve: 21; 66.3±6.1 y  45 samples (EC and adjacent myometrium) | STMN1 associated with poor survival |

Footnotes and abbreviations: (1) qRT-PCR: quantitative real-time PCR; WB: western blotting. (2) BMI: body mass index - kg/m^2^; y: years; EH: endometrial hyperplasia; EEC: endometrioid EC; SEC: serous EC; CCEE: clear cell EC. (3) 95% CI: 95% confidence interval; vs: *versus*; SN: sensitivity; SP: specificity; PPV: positive predictive value; NPV: negative predictive value; AUC: area under the curve; ROC: receiver operating characteristic; DEP: differentially expressed proteins.

**Supplementary Table S8**: Metabolomics studies in endometrial cancer.

| **Study**  **Aim** | **Samples** | **Study design**  **Verification** | **Method** | **Control group** ^(1)^ | **Case group** ^(1)^ | **Findings** | | |
| --- | --- | --- | --- | --- | --- | --- | --- | --- |
| **Blood (plasma or serum) samples** |  |  |  |  |  |  | | |
| Ihata, 2014 (Ihata, Miyagi et al. 2014)  Diagnostic Biomarkers | Plasma | Case-control | HPLC-ESI-MS  Targeted | N= 362  benign gynecological diseases (n=122); 45y (32-82y)  Leiomyoma: 54  Adenomyosis: 7  Endometrial cyst: 18  Cystic teratoma: 14  Mucinous cyst: 13  Serous cyst adenoma: 1  Fibroma: 4; Simple cyst: 9  Others: 2  Healthy women (n=240  Age and BMI matched  58y (32-82y)  Training set: 120 healthy women  Validation set (n=242)  120 healthy women + 122 patients with benign diseases | N=80  EC patients; 58y (32-80y)  Sage I: 48; Stage II: 9  Stage III: 15; Stage IV: 8  Grade 1: 40; Grade 2: 15  Grade 3: 6; Unknown: 19  EEC: 54; SEC: 6; CCEC: 3  Adenosquamous EC: 6  Mucinous: 1  Carcinosarcoma: 1  Squamous EC: 1  Poorly differentiated EC: 1  Training set: 40 EC patients  Validation set: 40 EC patients | Training:  EC vs Healthy women:  🡫 His, Trp, Val, Phe, Asp, Ser, Leu, and Met  🡩 ornithine, Ile, Pro  **LR models**:  EC vs Healthy women  His, Ile, Val and Pro:  AUC: 0.94; SN: 60%; SP: 98.3%  CA-125: AUC: 0.80  Validation set  EC vs Benign disease  His, Ile, Val, and Pro: AUC:0.83  CA-125: AUC: 0.60  EC stage I vs Healthy women  His, Ile, Val, and Pro: AUC: 0.91  CA-125: AUC: 0.79  EC stage II-IV vs Healthy women  His, Ile, Val, and Pro: AUC: 0.99  CA-125: AUC: 0.83 | | |
| Knific, 2018 (Knific, Vouk et al. 2018)  Diagnostic & Prognostic Biomarkers | Plasma | Case-control  Training and test sets  selected by splitting into equal sets 100 times | FIA-ESI-MS/MS  Absolute*IDQ*^TM^ p150 kit (Biocrates Life Sciences)  Targeted | N=65  Patients with benign pathologies  prolapsed uterus or myoma  63.2±9.4y; BMI: 28.3 ±4.7 | N=61  EC patients  65.1±8.7y; BMI: 32.1±7.3  LVI^-^ or unknown: 52  LVI^+^: 9; MI>½: 16  no difference between groups in age, menopausal status, medication intake, diabetes, hypertension, smoking status | 🡫 3 metabolites: PCaa C40:1, PCaa C42:5, PCaa C42:6, 166 metabolite ratios  🡩 total short-chain and long chain acylcarnitines, Pro/Tyr  LR model  EC vs controls  C16/PCae C40:1, Pro/Tyr, PCaa C42:0/PCae C44:5  AUC: 0.84; SN: 85.3%; SP: 69.2%  C16/PCae C40:1, Pro/Tyr, PCaa C42:0/PCae C44:5, smoking  AUC: 0.86; SN: 77.0%; SP: 79.0%  Detection of MI:  SMOH C14:1/SMOH C24:1, PCaa C40:2/PCaa C42:6  AUC: 0.86; SN: 81.3%; SP: 86.4%  SMOH C14:1/SMOH C24:1, C16:2/lyso PCa C16:1  AUC: 0.84; SN: 75%; SP: 72.7%  SMOH C14:1/SMOH C24:1, PCaa C40:2/PCae C40:1  AUC: 0.85; SN: 68.8%; SP: 97.7%  SMOH C16:1/SMOH C24:1, PCaa C34:4/PCae C34:3  AUC: 0.85; SN: 81.2%; SP: 77.3%  Detection of LVI:  PCaa C34:4/PCae C38:3, C16:2/PCaa C38:1  AUC: 0.94; SN: 88.9%; SP: 84.3% | | |
| Strand, 2019 (Strand, Tangen et al. 2019)  Prognostic Biomarkers | Plasma | Cases only  Cross-validation | Targeted  LC-MS/MS  Absolute*IDQ*^TM^ p180 kit  (Biocrates Life Sciences) | - | N=40  Group 1, short survival: 20  75y (63.6-81.5y)  EEC: 8; SEC: 5  Carcinosarcoma: 5  Non-endometrioid: 2  Grade 1: 3; Grade 2: 2  Grade 3: 3; Stage I: 18  Stage II: 2  Group 2, long survival: 20  67y (56.0 -77.y)  EEC: 7; CCEC: 3; SEC: 3  Carcinosarcoma: 6  Non-endometrioid: 1  Grade 1: 3; Grade 2: 2  Grade 3: 2; Stage I: 18  Stage II: 2  Group 1/2 matched histology, grade, age, BMI | Long/short survival:  🡫 methionine sulfoxide (MetSO), hydroxypropionylcarnitine (C3-OH)  **Model 1**: AUC: 0.82 (MetSO, serotonin, spermine, C3-OH, PCaa C36:5, SM C20:2)  **Model 2:** AUC: 0.935 (MetSO, serotonin, spermine, C3-OH, PCaa C36:5, SM C20:2, spermidine, butenylcarnitine (C4:1), lyso PCa C18:2 and lyso PCa C24:0)  **Model 3**: AUC: 0.965 (MetSO, serotonin, spermine, C3-OH, PCaa C36:5, SM C20:2, spermidine, C4:1, lyso PCaa C18:2, lyso PCaa C24:0, Asp, dimethylarginine, hexose, PCae C30:1) | | |
| Njoku, 2021 (Njoku, Campbell et al. 2021)  Diagnostic Biomarkers  Prognostic Biomarkers | Plasma | Case-control  study  Patients with BMI > 30  RF based on training and test sets | Non-targeted  Metabolon Inc ®  RP UHPLCMS/M; HILIC | N=69  Control patients referred to weight loss management  Normal histology  Postmenopausal: 21  46y (IQR 39.53y)  BMI: 50 (IQR 46.55) | N=67  EC patients  Postmenopausal: 56  Grade 1: 47; Grade 2: 14  Grade 3: 6; Stage I: 59  Stage II: 2; Stage III:6  LVI+: 12; >50% MI: 12  63y (IQR 54.69y)  BMI: 40 (IQR 34.46) | EC vs Controls  **Univariate ROC analysis**  1-lignoceroyl GPC (24:0),  AUC: 0.91  1-(1-enyl-stearoyl)-2-linoleoyl-GPE (P-18:0/18:2), AUC = 0.85  1-linolenoyl-GPC (18:3)  AUC: 0.84  3-hydroxybutyryl carnitine,  AUC:0.83  3-hydroxybutyrate  AUC; 0.82  **RF algorithm**  EC vs Controls (top 20 metabolites)  Training (86% accuracy),  *Test set* (93% accuracy)  AUC: 0.95  EC stage I vs Controls (top 20 metabolites); AUC: 0.98  EC with/without LVI: AUC=0.832 (1-linoleoyl-GPE)  EC with/without > 50% MI: AUC=0.74 (Homovanillate)  -only postmenopausal women: AUC=0.83 (Tricosanoyl sphingomyelin) | | |
| Kliemann, 2021 (Kliemann, Viallon et al. 2021)  Association | Plasma (EPIC)  serum (Intercept) | Nested case-control study  (EPIC, Intercept)  Discovery metabolic signatures of BMI, waist circumference (WC) and waist/hip ratio (WHR)  Validation associations with EC | Targeted LC-MS/MS  Absolute*IDQ* p180 kit | N=648  Control patients  53.99±7.9y; BMI: 25.9±4.2  Matched for study recruitment center, age, menopausal status, time of blood collection, fasting status,  No diabetes, no HRT | N=635  EC patients  53.96±7.9y; BMI: 28±5.4 | Metabolic signatures of body size (BMI, WC and WHR)  🡩 Val, Ile, Gln, PCaa C38:3, PCaa C38:4,  🡫 Asn, Gln, Gly, Ser, lysoPC C17:0, lysoPC C18:1, lysoPC C18:2, PCaa C42:0, PCaa C34:3, PCaa C40:5, PCaa C42:5  EC associated with metabolic signatures of BMI, WC and WHC  (Conditional LR models)  BMI OR 1.5 (1.30-1.74)  WC OR 1.46 (1.27-1.69)  WHC OR 1.54 (1.33-1.79) | | |
| Dossus, 2021 (Dossus, Kouloura et al. 2021)  Association | Plasma | Nested case-control study  (EPIC) | Targeted LC-MS/MS  Absolute*IDQ* p180 kit | N=853  Control patients  54.7±7.5y; BMI: 25.7±4.1 | N=853  EC patients  Type 1: 761  Type 2: 42  Not known: 50  54.7±7.5y; BMI: 27.7±5.4 | (Conditional LR models)  28 metabolites associated with EC (12 GP, 2 acylcarnitines and 2 sphingolipids)  Adjustment for BMI:  SM C18:0: OR 1.18 (1.05-1.33)  Gly, Ser, free carnitine (C0):  OR 0.89 (0.80-0.99)  OR 0.89 (0.79-1.00)  OR 0.91 (0.81-1.00)  Esterified/free carnitine OR 1.14 (1.02-1.28)  Short chain/free acylcarnitines OR 1.12 (1.00-1.25) | | |
| Breeur, 2022 (Breeur, Ferrari et al. 2022)  Association | Plasma and serum samples from EPIC study | Case-control study  Pan- cancer study  with 11 656 participants | Targeted metabolomics  LC-MS/MS  Absolute*IDQ* p150, Absolute*IDQ* p180 | N = 689  Control women:  54.3 ±7.8y; BMI: 26.0 ±4.3 | N = 689  EC patients:  54.3 ±7.8y; BMI: 28.2 ±5.5  Cases were matched with controls with regard to study centre, sex, age, time of the collection, fasting status, and exogenous hormones. | Different cancers (BC, colorectal, prostate cancer/control women) 6 metabolites: 🡫 Gln, butyrylcarnitine (C4), lysoPCa C18:2, PCaa 32:2, PCaa 36:0, PCaa 36:1,  🡩 Pro, decanoylcarnitine (C10), PCaa C28:1  EC/control women  🡩 SM_C16:0 cluster OR 1.51 (1.19-1.93)  OR 1.20 (0.97-1.47) adjusted for additional factors | | |
| Audet-Delage, 2018 (Audet-Delage, Villeneuve et al. 2018)  Discovery  Diagnostic  prognostic | Serum | Case-control  exploratory | Metabolon platform  RP-UPLC-MS/MS  Non-targeted | N=18  Patients with benign conditions  postmenopausal  no HRT for the last 3 weeks  58.9±10.4y; BMI: 27.5±7.2 | N=26  EC patients  Type 1: 24; Type 2: 12  postmenopausal  no HRT for the last 3 weeks  non-recurrent (NR) N= 18  Type 1: 12; Type 2: 6  66.3±8.3y; BMI: 28.4±7.0  recurrent (R) N = 18  Type 1: 12; Type 2: 6  67.5±9.4y; BMI: 28.0±6.4 | 1592 metabolites analysed,  EC/C: 137 metabolites, 🡩115 (acylcholines, monoacylgycerols, acylcarnitines), 🡫22 (free fatty acids)  Peptides and amino acids: spermine and isovalerate, glycylvaline, gamma-glutamyl-2-aminobutyrate AUC = 0.92 (0.84-1.00)  Type 1/type 2: 98 metabolites, 🡩 30 (bradykinin, sulfated androgens)  🡫 68 (heme, saturated long-chain acylcarnitine, choline, sarcosine, Gly)  R/NR: 104 metabolites (80 involved in lipid metabolism)  🡩 monoacylglycerols (16:1, 18:1, 20:5 and 22:6), docosahexaenoyl carnitine, 2-hydroxypalmitate, 2-hydroxystearate 🡫 Ser, Thr  R/NR: 2-oleoylgycerol and TAG 42:2-FA12:0  AUC = 0.90 (0.79-1.00)  Type 1 R cases 🡫 bile acids (taurodeoxycholate, glycodeoxycholate and taurocholate) 🡩 phosphorylated fibrinogen cleavage peptide  Type 2 R cases 🡩 sphingolipids (ceramides, dihydroceramides, lactosylceramides) | | |
| Audet-Delage, 2018 (Audet-Delage, Grégoire et al. 2018)  Discovery  Prognostic  Diagnostic | Serum  pre-surgical &  one month after surgery | Case-control  Cases only  Training only | Targeted  GC-MS (13 unconjugated steroids), RP-LC-MS/MS (14 conjugated steroids, catechol estrogens) | N=110  healthy postmenopausal women,  58.3±5.6y  OC: 145 no, 91 yes, 10 missing  HRT: 157 never, 80 ever, 9 missing | N=246  EC patients  65.1±8.9y  Type 1: 202; Type 2: 44  Grade 1: 90; Grade 2: 94  Grade 3: 61; Unknown: 197  Stage I: 197; Stage II: 12  Stage III: 28; Stage IV: 9  MyomInv<50%: 187  MyomInv>50%: 59  LVI^-^: 183; LVI^+^: 58  No relapse: 220  Relapse: 26  5 year recurrence: 24  (FW time: 65.5m)  OC: 19 no, 91 yes  HRT: 40 never, 70 ever | BMI: 🡩 E3, E1-S, E1, E2, 2MeO-E1  MI 🡫 E3  Recurrence (pre-operative):  🡩 E1-S  🡫 E3  EC (after)/EC (before): 🡫 all steroids except 🡩 4MeO-E2  EC (after) ≈ HW¸🡩 4MeO-E2  EC (type 1 and type 2 before) vs healthy  🡩 DHEA-S, DHEA, 5-diol,4-dione, testosterone, DHT, ADT-G, 3a-Diol-G, 3a-Diol-17G, E1-S, E1, E2  EC (type 2, before) vs healthy  🡩 DHEA, 5-diol, 4-dione, testosterone, ADT-G | | |
| Troisi, 2018 (Troisi, Sarno et al. 2018)  Discovery  Validation  Diagnostic  Prognostic | Serum | Case-control  Recruitment I  Recruitment II | Non-targeted  GC-MS  kit (Theoreo) | N=130  1st group:  80 Healthy Women  60y (55-65y); BMI: 27.8 (24.2-29.0)  2nd group:  50 Healthy Women  65y (59-69y); BMI: 27.1 (23.9-30.5)  10 Patients with benign diseases (hyperplasia, polyps, abnormal bleeding) | N=128  1st group: n=88  Type I: 67; Type II: 21  Grade 1: 2; Grade 2: 53  Grade 3: 33; Stage I: 36  Stage II: 45; Stage III: 7  68y (62-68y); BMI: 28.3 (25.1-30.3)  2nd group: n=30  Type I: 23; Type II: 7  Grade 1: 4; Grade 2: 22  Grade 3: 4; Stage I: 12  Stage II: 15; Stage III: 3  66y (61-72y); BMI 28.9 (26.3-31.1) | 259 metabolites determined consistently  Ensemble models  PLS-DA model (also LDA, NB, DT, RF, K-NN, ANN, SVM)  EC/HW (Model I):  🡩 lactic acid, homocysteine, 3-hydroxybutyrate  🡫 linoleic acid, stearic acid, myristic acid, Thr, Val, progesterone  Accuracy: 0.99±0.0  SN: 97±3%; SP: 98±2%  type 1/ type 2 (Model II):  🡩 lactic acid, Cys, Ser, malate, Glu, homocysteine,  🡫 progesterone  Accuracy: 0.93±0.04  SN: 96±4%; SP: 86±13%  Ensemble models I and II  Accuracy: 1.00 ±0.0  SN: 100 ±0.0 %; SP: 100 ±0.0 % | | |
| Shi, 2018 (Shi, Wang et al. 2018)  Discovery  Exploratory | Serum | Case-control  Cross validation with 100 random permutations | RP-UPLC-ESI-Q-TOF/MS  Non-targeted | N=46  Healthy women  57 ±10y; BMI 25.8±3.1 | N=46  Type 1 EC  Stage Ia: 27; Stage IIb: 19  Grade 1: 20; Grade 2: 13  Grade 3: 13  52 ±8y; BMI 26.9±5.1 | PLS-DA and OPLS-DA model:  7646 in positive mode, 2579 negative mode  🡩 Phe, indoleacrylic acid, phosphocholine (PC), lyso-platelet-activating factor 16 | | |
| Bahado-Singh, 2017(Bahado-Singh, Lugade et al. 2017)  Discovery (Training + test set)  Validation  Diagnostic | Serum | Case –control  random stratification into test and training | Non-targeted  NMR (32)  Targeted  Absolute*IDQ*^TM^  RP-LC-MS/MS (149)  (Biocrates Life Sciences) | N=60  Healthy women  59.2±12.7y  Training + test set: 36  Validation set: 24 | N=56  59.1±12.8y  Stage I-II: 46; Stage III-IV: 10  Training + test sets: 33  Validation set: 23 | All EC/HW  Significant differences: 4/32; 36/149 (16 overlap)  VIP: 3-hydroxybutyrate, C14:2, C6 (C4:1DC), C10, C18:2, L-Met, C8, 2-hydroxybutyrate, C7-DC, C18:1, C16, kynurenine, C14:1, PCae C40:1  LR model (validation data)  EC/HW  C14:2, PCae C38:1, 3-hydroxybutyric acid  AUC: 0.83 (0.70-0.95)  SN: 82.6%; SP: 70.8%  C18:2, PCae C40:1, C6, C4:1-DC  AUC: 0.81 (0.69-0.94)  SN: 82.6%; SP: 66.7%  BMI, C14:2, PCae C40:1  AUC: 0.80 (0.67-0.78)  SN: 78.3%; SP: 62.5%  EC stage I-II/ HW  PCae C38:1, 3-hydroxybutyric acid, C14:2  AUC: 0.82 (0.69-0.95)  SN: 72.2%; SP: 79.2%  BMI, C14:2, PCae C40:1  AUC: 0.80 (0.67-0.93)  SN: 72.2%; SP: 75.0% | | |
| Lunde, 2020 (Lunde, Nguyen et al. 2020)  Exloratory study  Prediction of postoperative pain | Serum  Danish Cancer Biobank | Cases only  From a nested case-control study  reference test: hysterectomy,  prognosis of pain according to VAS > 3  five-fold cross-validation | Non-targeted NMR | - | N=78  Low-risk: 50  Intermediate-risk: 9  High-risk: 19  With chronic postoperative pain: 26  Without pain: 52  matched on age and BMI | 🡩 19 metabolites  branched-chain amino acids, cholesterol, cholesteryl ester, linoleic acid, phospholipids, triglycerides  🡫 glycerol  EC with/without pain  IDL-TG, LDL-TG, L-LDL-TG, AUC = 0.8  PLS-DA, LSVM, LR, RF models including 14 metabolites:  AUC = 0.79-0.87 | | |
| Kozar, 2021 (Kozar, Kruusmaa et al. 2021)  Discovery | Serum | Prospective observational study  Cross validation with 50 iterations (training and test set) | Non-targeted  HPLC-TQ/MS | N=21  Control patients (pelvic floor disorders, endometriosis, benign ovarian cysts)  Premenopausal: 15 Postmenopausal: 6  54± 19 y  BMI:25±4 | N = 15  EEC patients  Premenopausal: 3  Postmenopausal: 12  stage I: 9  stage II: 2  stage III: 3  stage IV: 1  Grade 1: 10  Grade 2: 4  Grade 3: 1  Age: 64 ± 14 y  BMI: 29± 7 | EC/C  Univariate analysis  Cer 34:1;2  Cer 40:1;2  AC 16:1-OH; 1-methyladenosine, AC 16:1, AC 14:1; AC 14:0  AUC = 0.75-0.83; SEN: 60-86%, SP: 73-95%, accuracy: 0.22-0.81  RF model (Cer 34:1;2, Cer 40:1;2, AC 16:1-OH and 1-methyladenosine  AUC = 0.92 (0.91-0.95); SEN: 94%, SP: 75%, accuracy: 22-81% | | |
| Gu, 2021 (Gu, Chen et al. 2021)  Discovery  Diagnostic  Prognostic | Serum | Case-control  Only training | Non-targeted  GC-MS | N=30  postmenopausal patients indicated hysterectomy  No HRT (3 weeks before)  40-85 y | N=60  Postmenopausal  no HRT (3 weeks before)  Stage I: 30  Stage II: 30  40-85 y | EC stage I/C: 27 metabolites: 7🡩, 20🡫  OPLS-DA  (VIP> 1.5): urea, arachidonic acid, mannose, phosphoric acid, threose, GABA, 1-monopalmitoylgycerol, ethylamine, cholesterol  EC stage II/C: 28 metabolites: 6🡩, 22🡫  (VIP> 1.5): diphosphate, 3-oxaoct-4-en-11-imine, β-D-allopyranose, Ser, L-Ile, Gly, arachidonic acid, 1-monopalmitoylglycerol, GABA, aminomalonic acid, oxalic acid, urea  EC stage I/stage II: 25 metabolites 7🡩, 18🡫  (VIP> 1.5): D-galactose, phosphoric acid, threose, urea, 5-hydroxycaproic acid, cholesterol, mannose, GABA, β-D-allopyranose | | |
| Yan, 2022 (Yan, Zhao et al. 2022)  Discovery  and validation  Diagnostic  Prognostic | Serum | Case-control  Discovery (95)  validation (456) | Non-targeted  UPLC-Q-TOF/MS | N=496  Discovery (n=72)  Healthy women: 30  51.9 ±7.5 y  Premonopausal: 13 Postmenopausal: 11  Perimenopausal: 6 (stratification according to age)  Endometrial polyps: 30  51.9 ±10.9 y  Postmenopausal: 16  EH: 12  46.8 ±8.4 y  Postmenopausal: 1  No HRT (3 months before)  Validation (n=406)  Healthy women: 195  Endometrial polyps: 171  EH: 40 EH | N=73  Discovery (n=23)  58.7 ±8.6 y  EEC: 20  SEC: 2  Muellerian: 1  Grade 1: 7  Grade 2: 6  Grade 3: 3  Unknown: 7  Stage Ia: 11  Stage Ib: 5  Stage II: 1  Stage III: 3  Stage IV: 2  LVI-: 7  LVI+: 9  Postmenopausal: 21  No HRT 3 months before surgery, no info about BMI  Validation: 50 EC  No other data | EC/HW  OPLS-DA model (VIP>1, p< 0.05) discovery + validation:  🡩 LysoPC 20:2, Lyso PC 20:4,  🡫DG 38:5; Cer(d18:0/18:0), PG 34:0, CE 16:0, PC 38:3, PC 15:1  AUC: 0.737-0.882, SEN: 64%-92%, SP: 56.5%-91.3%  Logistic regression model (4 EP specific biomarkers: 6-ketoPGF1α, PA 37:4, LysoPC 20:1, PS 36:0)  EP/EC (discovery/validation):  AUC = 0.92, SEN: 100%, SP: 72.4%  AUC = 0.90, SEN: 70.4%, SP: 94.1%  EC stage I /EP  AUC = 0.90, SEN: 94.8%, SP: 76.9% | | |
| Schuhn, 2022 (Schuhn, Tobar et al. 2022)  Discovery  Diagnostic | Serum | Case-control  Training only | Targeted  17 amino acids and 28 acylcarnitines  ESI-MS/MS | N=171  Healthy women: 157  48 ±14 y  BMI: 26.0±7.1  N= 14  Patients with benign pathologies (polyps, suspicious endometrium on imaging):  56 ±12 y  BMI: 28.0 ±7.6 | N=20  EC patients  62 ±9 y  BMI: 30 ±6.2 | EC/C:  🡫Thr, Arg, Met, 🡩 malonylcarnitine  AUC = 0.73-0.85  Thr  AUC = 0.85 (0.72-0.98), SEN: 70%, SP: 92.9%  EC/HW:  malonylcarnitine, acetylcarnitine, carnitine, tetradecenoylcarnitine  AUC = 0.75-0.82  malonylcarnitine  AUC = 0.82 (0.72-0.92), SEN: 80%, SP: 73.1% | | |
| Dried blood samples | | |  |  |  |  | | |
| Troisi, 2020 (Troisi, Raffone et al. 2020)  Discovery and validation  Diagnostic | Dried blood samples | A multicenter prospective cohort study  discovery and validation | Non-targeted GC-MS | N= 70  Discovery  Patients without EC (matched age, years from menopause, tobacco use, comorbidities) postmenopausal, no HRT, no previous hysterectomy, no immunosuppressive therapy  Age: 68.2 ± 11.7 y  BMI: 27.6 ± 4.3  N = 1430  Validation:  Prospective cohort of postmenopausal women (16 women were diagnosed with EC, see next cell) | N = 50  Discovery  EC (postmenopausal, FIGO I-III, G1-G3)  N=16 EC (from the prospective cohort of 1430 subjects)  Age: 69.4 ± 13.8 y  BMI 29.3± 4.9  12 IA, 3 IB, 1 II  Age: 59.7± 7.7 y  BMI: 26.8± 4.6 | EC/C  Classification models:  Decision tree, Naive Bayes, RF, k-Nearest neighbours, Artificial neural network, Linear discriminating analysis, SVM, linear regression, Deep Learning, Partial least squares-discriminant analysis  SP: 96.3-100%, SEN: 50- 100 %; PPV: 89.7-100%, NPV: 80-100%, Accuracy: 83.3-100  Ensemble Machine Learning algorithm (10 different classification models  SP: 99.9%, SEN: 100 %; PPV: 88.9 (7.4), NPV: 100%, Accuracy: 99.9 % | | |
| Urine samples | | |  |  |  |  | |  |
| Shao, 2016 (Shao, Wang et al. 2016)  Discovery  Diagnostic | Urine  (morning) | Case-control  Training and test set | RP-UPLC-ESI-Q-TOF-MS  Non-targeted | N=35  Healthy women (n=25)  EH (n=10) | N=25  EC patients  no significant difference in age and weight | PLS-DA model (all 60 patients)  5 metabolites EC/HW+EH  🡫 porphobilinogen, acetylcysteine  🡩 N-Acetylserine, urocanic acid, isobutyrylglycine  SVM model  EC/HW+ EH (2/3 training set, 1/3 test set) | | |
| Cervicovaginal fluid | | |  |  |  |  | | |
| Cheng, 2019 (Cheng, Chen et al. 2019)  Discovery  Diagnostic | Cervicovaginal fluid  Collected in the middle of the menstrual cycle. | Case-control  NCT02528864  Training and test set | Non-targeted  ^1^H NMR  Bruker Advance 600 MHz | N=33  Patients with benign pathologies  47 y (32-74 y)  Fibroid: 17  Endometrioma: 7  Adenomyosis: 5  Polyp: 4  Pre-menopause: 26  Post-menopause: 7  Cases/controls: no differences in diabetes, metabolic syndrome, undergoing estroprogestinic therapy | N=21  EC patients  52 y (30-67 y)  Stage I: 17  Stage II: 1  Stage III: 3  Grade 1 or 2: 12  Grade 3: 7  Pre-menopause: 13  Post-menopause: 8 | EC/C  Training data set: 17 cases, 28 controls  Test data set: 4 cases; 5 controls  29 metabolites identified  Significant 🡩: choline, formate, fumarate, malate, phosphocholine  Significant 🡫: Asn, Asp, Ile, Phe, pyruvate  Prediction models built upon phosphocholine, malate, Asn  EC/C  Training:  RF: AUC = 0.92 (0.80-0.99)  SVM: AUC = 0.88 (0.76-0.97)  PLS-DA: AUC = (0.89 (0.76-0.97)  LR: AUC = 0.88 (0.70-0.97)  ANN: AUC = 0.88 (0.82-0.92)  Testing:  RF: Acc. 0.78 (0.4-0.97); SEN 0.75 (0.19-0.99); SP. 0.8 (0.28-1.00)  SVM: Acc. 0.78 (0.4-0.97); SEN. 0.75 (0.19-0.99); SP. 0.8 (0.28-1.00)  PLS-DA: Acc. 0.67 (0.3-0.93); SEN 0.75 (0.19-0.99); SP 0.6 (0.15-0.95)  LR: Acc. 0.67 (0.3-0.93); SEN 0.75 (0.19-0.999; SP 0.6 (0.15-0.95)  ANN: Acc. 0.73 (0.63-0.8); SEN. 0.68 (0.55-0.74); SP0.64 (0.52-0.72) | | |
| Tissue samples | | |  |  |  |  | | |
| Jove, 2016 (Jove, Gatius et al. 2016)  Discovery  Diagnostic | Tissue | Case-control  (permutation test) | RP-LC-ESI-QTOF-MS/MS  Non-targeted | N=15  Normal endometrium  Proliferative (n=10)  Secretory (n=10) | N=27  EEC  Grade 1: 6  Grade 2: 13  Grade 3: 8  Two different samples:  Surface endometrioid carcinoma (SEC)  myometrial invasive front (MIF) | EC/NE:  44 metabolites (4 identified)  🡩 stearamide, monoolein,  hypoxanthine, 1,2-dihexadecanoyl-sn-glycerol  PLS-DA  G III-IV/I-II:  26 metabolites (3 identified)  🡫 taurine, erythriol,  🡩 oleamide  SEC/MIF  104 metabolites (14 identified):  🡩 xanthine, lactamide, lactic acid, alpha-D-fucose, 3-mercaptopyruvate, ribitol, PC 32:0, eicosapentaenoic acid  🡫 inosine, deoxycytidine, hypoxanthine, CDP-ethanolamine, 5-methylthioadenosine, monoolein | | |
| Altadill, 2017 (Altadill, Dowdy et al. 2017)  Discovery  Diagnostic  Prognostic | Tissue | Case-control  Training only | Non-targeted  RP-UPLC-ESI-TOF-MS  Waters SYNAPT G2 Si | N=17  Benign diseases  > 50 y  Postmenopausal  no treatment | N=39  Stage Ia: 10  Stage Ib: 9  Stage II: 10  Stage III: 10  >50 y  Postmenopausal  no treatment | EC vs Controls  80 metabolites, 42 identified mainly lipids 🡩 8 glycerophosphocholines (PC), 1 phosphatidylserine (PS), 1 phosphatidylglycerol (PG), 9 phospatidylethanolamines (PE), 4 phosphatidylinositol (PI); linoleic acid, 3-deoxyvitamin D3, UDP-N-Acetyl-D-galactosamine, 1-palmitoyl-2-linoleoyl PE  🡫 Glu-Phe-Arg-Trp, palmic amide, stearamide, oleamide, 1 PA, 2 PE, PG, inosine, picolinic acid  29 stage I/II EC vs 10 stage III  🡩 PC (16:0/20:5), PE (22:6/P-18:1), UDP-N-acetyl-D-galactosamine, arachidonic acid, 🡫 PC (16:0/22:6), 2 PE (16:0/22:6), (18:1/22:6) | | |
| Trousil, 2014 (Trousil, Lee et al. 2014)  Discovery  Diagnostic | Tissue | Case-control  (Seven fold cross-validation) | ^1^H-NMR  Bruker DRX600  Non-targeted | N = 10  Normal endometrium  Median age 47.8 y | N = 10  EC patients G3  Median age 65.8 y | EC/C  🡩 Val, Leu, Ala, Pro, phosphocholine, Tyr  🡫 glutathione, scyllo-inositol, myo-inositol, inosine/adenosine | PLS-DA model  AUC = 0.987 | |
| Cummings, 2019 (Cummings, Massey et al. 2019)  Discovery  Diagnostic | Tissue | Case-control  training | Targeted  RP-LC-MS/MS | N=53  Patients  undergoing hysterectomy  Proliferative: 13  Secretory: 6  Atrophic: 33  AEH: 31 | N=108  67 y (39-89 y)  Stage I: 79  Stage II: 7  Stage III: 14  Stage IV: 8 IV  LVI^-^: 58  LVI^+^: 50  Type 1: 55  Grades 1 and 2  Type 2: 53  Grade 3: 10  SEC: 19  CCEC: 5  Mixed EC: 4  Carcinosarcoma: 15 | EC/C  Dihydro-15-keto derivatives:  🡫 type 1 and type 2 /NE  13,14-dihydro-15-keto PGE2  🡫 type 2 /NE  13,14-dihydro-15-keto PGF2α  Type 2/ type 1 EC:  🡫 12-HETE | | |
| Skorupa, 2021 (Skorupa, Ponski et al. 2021)  Discovery  Diagnostic | Tissue | Case-control  (Seven fold cross-validation) | Non-targeted  HR MAS NMR | N=10  Patients with benign diseases (prolapse of uterus, leiomyomas, cystadenomas)  62.0±7.3 y  BMI: 27.0±2.2  No HRT | N=64  Grade 1: 14  64.9±9.1 y  BMI: 26.9±2.0  Grade 2: 33  70.2±7.4 y  BMI: 30.5±5.6  Grade 3: 17  68.0±8.4 y  BMI: 29.4±3.3  No HRT | OPLS-DA  EC/C: 🡩 Val, Leu, Ile, hypotaurine, Ser, Lys, ethanolamine, choline  🡫 creatine, creatinine, glutathione, ascorbate, Gln, PE, scyllo-inositol  EC G1/2/C: 🡩 taurine  EC G1/3/C: 🡩 Gly, N-acetyl compound, lactate  G1: 🡩 dimethylsulfone, phosphocholine, 🡫 glycerophosphocholine, Gln  G2/3 🡫 myoinositol  G3: 🡩 3-hydroxybutyrate, Ala, betaine  G1/G2  Dimethyl sulfone AUC = 0.95 (0.86-0.99)  G2/G3  Taurine AUC = 0.92 (0.84-0.98)  Scyllo-inositol AUC = 0.92 (0.83-0.99)  Choline AUC = 0.85 (0.73-0.94)  G1/G3  Choline AUC = 0.96 (0.86-1.0)  3-hydroxybutyrate AUC = 0.93 (0.81-1.0)  Taurine AUC = 0.95 (0.83-1.0) | | |
| Arda Düz, 2022 (Arda Düz, Mumcu et al. 2022)  Discovery  Diagnostic | Tissue | Case-control  Statistical validation (100 times permutation) | Nontargeted  ^1^H HR-MAS | N = 18  Patients with benign diseases  Age: 49 ± 7.1 y  (cystadenoma, prolapse) hysterectomy performed, histopathological normal endometrium  No HRT  Matched in terms of age, BMI, menopausal status | N = 17  EC patients  Age: 53.5 ± 7.9 y  No HRT  Matched in terms of age, BMI, menopausal status  No data about Grade, FIGO stage | EC/C  PLS-DA models  (VIP>1, p< 0.05)  🡩lactate, Ala, choline/glycerophosphocholine/phosphocholine/O-phosphoetanolamine, Glu/Gln/Met, taurine, Glu, Leu/Ile, O-phosphoethanolamine, Ile ,Val, Gln, N-acetyltyrosine/Arg, Arg/Leu  AUC for individual metabolites/combinations > 0.85  Lactat: AUC= 0.88  Glu/Gln/Met: AUC= 0.87  Ala: AUC= 0.86  Phe: AUC= 0.85  Leu/Ile: AUC= 0.85 | | |
| Gatius, 2022 (Gatius, Jove et al. 2022)  Discovery  Prognosis | Tissue from Biobank | Cases only  training | Non-targeted LC-MS/MS  LC-ESI-Q-TOF MS/MS |  | N = 31  EEC = 20  Grade 1: 6  Grade 2:12,  Grade 3: 2  SEC = 11  Grade 3 =11  Age: 39-86 y (3 not available)  Recurrence  EEC: 2  Serous EC: 6 | PLS-DA model (VIP > 1, p< 0.05)  EEC/serous EC: 232 different metabolites  metabolites: 🡫 in serous EC:  LysoPG 18:1, SM d43:2, SM d44:2, eicosadienoic acid, PA 48:0, tetracosatetraenoic acid, 3-hydroxypristanic acid  Metabolites 🡩 in serous EC: adenosine-monophosphate, 2'-deoxyguanosine-5'-monophospate | | |
| Different samples | | |  |  |  |  | | |
| Shafiee, 2020 (Shafiee, Ortori et al. 2020)  Discovery  Diagnostic | Plasma and  tissue samples | Cros-sectional study  PCOS based on Rotterdam criteria.  Training only | Non-targeted  LC-HRMS | N=68  PCOS: 34 patients  Secretory: 3  Proliferative: 23  Unknown: 8  31.8± 6.0 y  BMI: 29.3± 2.9  Control patients (uterine fibroids, benign ovarian cysts, tubal ligation): 34  Premenopausal: 20  Perimenopausal: 14  43.7± 13.1 y  BMI: 28.6± 2.6  similar WHR no HT | N=34  Premenopausal: 4  Perimenopausal: 13  Postmenopausal: 17  Moderately differentiated: 44.1%  Poorly differentiated: 29.4%  Well differentiated: 26.5%  63.4±10.1 y  BMI: 32.2± 5.7 | PCOS/C and EC/C  no metabolite changed in blood samples  tissue samples:  🡫 monoacylglycerol 24:0 and capric acid  EC/C tissue samples  🡩 hydroxyundecanoyl carnitine, phoshorylcholine, diglyceride (26:4e). phosphatidylcholine (PC) 36:6; phosphatidylethanolamine (PE) 38:2; ceramide d29:2; PE 36:6e, diglyceride 36:6, ceramide d34:O, phosphatidylglycerol 36:2, acylcarnitine 17:0, monoacylglycerol (MAG) 18:2, PC 16:1e  🡫triglyceride 33:0; MAG 24:0, hexacosanoic acid, diacyglycerol 36:4, MAG 24:1, MAG 22:0, sterol (C27H48O5); MAG 22:4; oxotestosterone; triglyceride 28:0; MAG 22:2, MAG 24:4, DHT sulfate, triglyceride 24:0, capric acid | | |
| Yi, 2022 (Yi, Xie et al. 2022)  Discovery  Diagnostic | Tissue  Urine  intrauterine brushing samples | Case-control  Monte Carlo  Cross Validation | Nontargeted  ESI Q-TOF MC combined with LC-20A HPLC | N=43  Control patients (hysteromyoma, cyst, endometrial polyps and cervix diseases)  Tissue samples: 18  39.8 ± 5.3 y  BMI: 23.0± 2.5  All premenopausal  Urine samples: 10  44.3± 9.1 y  BMI; 22.4 ± 2.9  Postmenopausal: 25%  Intrauterine brushing samples: 11  48.2± 5.7 y  BMI: 22.6± 3.0  Postmenopausal: 18% | N=44 (all Type 1 EC)  Tissue samples: 24  48.2± 13years  BMI: 24.8 ± 3.9 kg/m2  Postmenopausal: 33.3 %  Grade 1: 24  Stage Ia: 22  Stage II: 1  Stage IIIa: 1  Urine samples: 10  44.0± 11.6 y  BMI. 27.1 ± 5.9  Postmenopausal: 30%  Grade 1: 9  Grade 2: 1  Stage Ia: 10  Intrauterine brushing samples: 10  45.8± 9.9 y  BMI: 24.8± 3.6  Postmenopausal: 20%  Grade 1: 9  Grade 2: 1  Stage Ia: 10 | PLS-DA models (VIP > 1, p< 0.05)  EC/C  tissue 74 metabolites  urine 285 metabolites  intrauterine brushing 122 metabolites  Urine (top 100 metabolites)  N-acetylaspartylglutamate, pseudouridine, adenosylmethionine, xanthine, His, hydroxykynurenamine  AUC = 0.81 (0.46-1.0) (100), 0.781 (0.40-1.0) (50)  Intrauterine brushing (100 metabolites)  Uridine, Ala, homoserine, Gln, N-acetylputrescine, formylkynurenine  AUC = 0.85 (0.58-1.09 (100), 0.82(0.52-1.0) (50)  Comparison tissue/urine/brushing  Tissue: 74 metabolites: 🡩 47, 🡫27  Urine: 49/74 metabolites: 🡩 6, 🡫43  Intrauterine brushing: 21/74 metabolites: 🡩 9, 🡫12 | | |

(1) BMI: body mass index - kg/m^2^.

**Abbreviations:** ANN, artificial neural network; DHEA, dehydroepiandrosterone; DHT, dihydrotestosterone; E1, estrone; E2, estradiol; E3, estriol; E1-S, estrone-sulfate; ESI-MS/MS, electrospray ionisation tandem mass spectrometry; G, grade; HRT, hormone replacement therapy; IDL, intermediate-density lipoproteins; LDL, low density lipoproteins; L-LDL, large LDL; LVSI, lymphovascular space invasion; MeO, methoxy; FW time: follow up time; MD, missing data; MI, myometrial invasion; NA, not available; ND, not determined; OPLS-DA, orthogonal partial least squares discriminant analysis; OC, oral contraception; OR, odds ratio; PCA, Principal Component Analysis; PCae, PCaa, glycerophospholipids; P, proliferative phase; PC, phosphatidylcholine, PE, phosphatidylethanolamine; PG, phosphatidylglycerol; PI, phosphatidylinositol; PLS-DA, Partial Least Squares Discriminant Analysis; RF, random forest; S, secretory phase; SEN, sensitivity; SM, sphingomyelin; SMOH, hydroxysphingomyelin; SP, specificity; SVM; support vector machine; TG, triglycerides; VIP, variable importance in projection; QTOF, quadrupole time of flight; y, years.

(10X-Genomics® http://www.10xgenomics.com/technology/)

**References**

- 10X-Genomics®. (<http://www.10xgenomics.com/technology/>). Retrieved accessed in December 2016.
- Aboulouard, S., M. Wisztorski, M. Duhamel, P. Saudemont, T. Cardon, F. Narducci, A. S. Lemaire, F. Kobeissy, E. Leblanc, I. Fournier and M. Salzet (2021). "In-depth proteomics analysis of sentinel lymph nodes from individuals with endometrial cancer." Cell reports. Medicine. **2**(6).
- Akkour, K., I. O. Alanazi, A. A. Alfadda, H. Alhalal, A. Masood, M. Musambil, A. M. Abdel Rahman, M. A. Alwehaibi, M. Arafah, A. Bassi and H. Benabdelkamel (2022). "Tissue-Based Proteomic Profiling in Patients with Hyperplasia and Endometrial Cancer." Cells **11**(13).
- Alonso-Alconada, L., M. Santacana, P. Garcia-Sanz, L. Muinelo-Romay, E. Colas, C. Mirantes, M. Monge, J. Cueva, E. Oliva, R. A. Soslow, M. A. Lopez, J. Palacios, J. Prat, J. Valls, C. Krakstad, H. Salvesen, A. Gil-Moreno, R. Lopez-Lopez, X. Dolcet, G. Moreno-Bueno, J. Reventos, X. Matias-Guiu and M. Abal (2015). "Annexin-A2 as predictor biomarker of recurrent disease in endometrial cancer." Int J Cancer **136**(8): 1863-1873.
- Altadill, T., T. M. Dowdy, K. Gill, A. Reques, S. S. Menon, C. P. Moiola, C. Lopez-Gil, E. Coll, X. Matias-Guiu, S. Cabrera, A. Garcia, J. Reventos, S. W. Byers, A. Gil-Moreno, A. K. Cheema and E. Colas (2017). "Metabolomic and Lipidomic Profiling Identifies The Role of the RNA Editing Pathway in Endometrial Carcinogenesis." Sci Rep **7**(1): 8803.
- Arda Düz, S., A. Mumcu, B. Doğan, E. Yılmaz, E. İnci Çoşkun, E. Sarıdogan, G. Tuncay and A. Karaer (2022). "Metabolomic analysis of endometrial cancer by high-resolution magic angle spinning NMR spectroscopy." Arch Gynecol Obstet.
- Attarha, S., S. Andersson, M. Mints and S. Souchelnytskyi (2013). "Individualised proteome profiling of human endometrial tumours improves detection of new prognostic markers." Br J Cancer **109**(3): 704-713.
- Audet-Delage, Y., J. Grégoire, P. Caron, V. Turcotte, M. Plante, P. Ayotte, D. Simonyan, L. Villeneuve and C. Guillemette (2018). "Estradiol metabolites as biomarkers of endometrial cancer prognosis after surgery." J Steroid Biochem Mol Biol **178**: 45-54.
- Audet-Delage, Y., L. Villeneuve, J. Grégoire, M. Plante and C. Guillemette (2018). "Identification of Metabolomic Biomarkers for Endometrial Cancer and Its Recurrence after Surgery in Postmenopausal Women." Front Endocrinol (Lausanne) **9**: 87.
- Bahado-Singh, R. O., A. Lugade, J. Field, Z. Al-Wahab, B. Han, R. Mandal, T. C. Bjorndahl, O. Turkoglu, S. F. Graham, D. Wishart and K. Odunsi (2017). "Metabolomic prediction of endometrial cancer." Metabolomics **14**(1): 6.
- Breeur, M., P. Ferrari, L. Dossus, M. Jenab, M. Johansson, S. Rinaldi, R. C. Travis, M. His, T. J. Key, J. A. Schmidt, K. Overvad, A. Tjonneland, C. Kyro, J. A. Rothwell, N. Laouali, G. Severi, R. Kaaks, V. Katzke, M. B. Schulze, F. Eichelmann, D. Palli, S. Grioni, S. Panico, R. Tumino, C. Sacerdote, B. Bueno-de-Mesquita, K. S. Olsen, T. M. Sandanger, T. H. Nost, J. R. Quiros, C. Bonet, M. R. Barranco, M. D. Chirlaque, E. Ardanaz, M. Sandsveden, J. Manjer, L. Vidman, M. Rentoft, D. Muller, K. Tsilidis, A. K. Heath, H. Keun, J. Adamski, P. Keski-Rahkonen, A. Scalbert, M. J. Gunter and V. Viallon (2022). "Pan-cancer analysis of pre-diagnostic blood metabolite concentrations in the European Prospective Investigation into Cancer and Nutrition." BMC Med **20**(1): 351.
- Celsi, F., L. Monasta, G. Arrigoni, I. Battisti, D. Licastro, M. Aloisio, G. Di Lorenzo, F. Romano, G. Ricci and B. Ura (2022). "Gel-Based Proteomic Identification of Suprabasin as a Potential New Candidate Biomarker in Endometrial Cancer." International Journal of Molecular Sciences **23**(4).
- Ceylan, Y., G. Akpinar, E. Doger, M. Kasap, N. Guzel, K. Karaosmanoglu, S. Y. Kopuk and I. Yucesoy (2020). "Proteomic analysis in endometrial cancer and endometrial hyperplasia tissues by 2D-DIGE technique." J Gynecol Obstet Hum Reprod **49**(2): 101652.
- Cheng, S. C., K. Chen, C. Y. Chiu, K. Y. Lu, H. Y. Lu, M. H. Chiang, C. K. Tsai, C. J. Lo, M. L. Cheng, T. C. Chang and G. Lin (2019). "Metabolomic biomarkers in cervicovaginal fluid for detecting endometrial cancer through nuclear magnetic resonance spectroscopy." Metabolomics **15**(11): 146.
- Cummings, M., K. A. Massey, G. Mappa, N. Wilkinson, R. Hutson, S. Munot, S. Saidi, D. Nugent, T. Broadhead, A. I. Wright, S. Barber, A. Nicolaou and N. M. Orsi (2019). "Integrated eicosanoid lipidomics and gene expression reveal decreased prostaglandin catabolism and increased 5-lipoxygenase expression in aggressive subtypes of endometrial cancer." J Pathol **247**(1): 21-34.
- DeSouza, L., G. Diehl, M. J. Rodrigues, J. Guo, A. D. Romaschin, T. J. Colgan and K. W. Siu (2005). "Search for cancer markers from endometrial tissues using differentially labeled tags iTRAQ and cICAT with multidimensional liquid chromatography and tandem mass spectrometry." J Proteome Res **4**(2): 377-386.
- DeSouza, L. V., J. Grigull, S. Ghanny, V. Dube, A. D. Romaschin, T. J. Colgan and K. W. Siu (2007). "Endometrial carcinoma biomarker discovery and verification using differentially tagged clinical samples with multidimensional liquid chromatography and tandem mass spectrometry." Mol Cell Proteomics **6**(7): 1170-1182.
- DeSouza, L. V., O. Krakovska, M. M. Darfler, D. B. Krizman, A. D. Romaschin, T. J. Colgan and K. W. Siu (2010). "mTRAQ-based quantification of potential endometrial carcinoma biomarkers from archived formalin-fixed paraffin-embedded tissues." Proteomics **10**(17): 3108-3116.
- Dossus, L., E. Kouloura, C. Biessy, V. Viallon, A. P. Siskos, N. Dimou, S. Rinaldi, M. A. Merritt, N. Allen, R. Fortner, R. Kaaks, E. Weiderpass, I. T. Gram, J. A. Rothwell, L. Lécuyer, G. Severi, M. B. Schulze, T. H. Nøst, M. Crous-Bou, M. J. Sánchez, P. Amiano, S. M. Colorado-Yohar, A. B. Gurrea, J. A. Schmidt, D. Palli, C. Agnoli, R. Tumino, C. Sacerdote, A. Mattiello, R. Vermeulen, A. K. Heath, S. Christakoudi, K. K. Tsilidis, R. C. Travis, M. J. Gunter and H. C. Keun (2021). "Prospective analysis of circulating metabolites and endometrial cancer risk." Gynecol Oncol **162**(2): 475-481.
- Enroth, S., M. Berggrund, M. Lycke, M. Lundberg, E. Assarsson, M. Olovsson, K. Stalberg, K. Sundfeldt and U. Gyllensten (2018). "A two-step strategy for identification of plasma protein biomarkers for endometrial and ovarian cancer." Clin Proteomics **15**: 38.
- Gatius, S., M. Jove, C. Megino-Luque, M. Alberti-Valls, A. Yeramian, N. Bonifaci, M. Pinol, M. Santacana, I. Pradas, D. Llobet-Navas, R. Pamplona, X. Matias-Guiu and N. Eritja (2022). "Metabolomic Analysis Points to Bioactive Lipid Species and Acireductone Dioxygenase 1 (ADI1) as Potential Therapeutic Targets in Poor Prognosis Endometrial Cancer." Cancers (Basel) **14**(12).
- Gu, M., X. Chen, Y. Sun, L. Wang, H. Shu and C. Qian (2021). "A metabolomic signature of FIGO stage I and II endometrial cancer." Neoplasma **68**(6): 1283-1291.
- Huang, H., Z. Hao, L. Long, Z. Yin, C. Wu, X. Zhou and B. Zhang (2021). "Dermatopontin as a potential pathogenic factor in endometrial cancer." Oncology Letters **21**(5).
- Ihata, Y., E. Miyagi, R. Numazaki, T. Muramatsu, A. Imaizumi, H. Yamamoto, M. Yamakado, N. Okamoto and F. Hirahara (2014). "Amino acid profile index for early detection of endometrial cancer: verification as a novel diagnostic marker." Int J Clin Oncol **19**(2): 364-372.
- Janacova, L., J. Faktor, L. Capkova, V. Paralova, A. Pospisilova, J. Podhorec, H. A. Ebhardt, R. Hrstka, R. Nenutil, R. Aebersold and P. Bouchal (2020). "SWATH-MS Analysis of FFPE Tissues Identifies Stathmin as a Potential Marker of Endometrial Cancer in Patients Exposed to Tamoxifen." Journal of Proteome Research **19**(7): 2617-2630.
- Janacova, L., J. Faktor, L. Capkova, V. Paralova, A. Pospisilova, J. Podhorec, H. A. Ebhardt, R. Hrstka, R. Nenutil, R. Aebersold and P. Bouchal (2617). "SWATH-MS Analysis of FFPE Tissues Identifies Stathmin as a Potential Marker of Endometrial Cancer in Patients Exposed to Tamoxifen." Journal of Proteome Research **19**(7): 2617-2630.
- Jove, M., S. Gatius, A. Yeramian, M. Portero-Otin, N. Eritja, M. Santacana, E. Colas, M. Ruiz, R. Pamplona and X. Matias-Guiu (2016). "Metabotyping human endometrioid endometrial adenocarcinoma reveals an implication of endocannabinoid metabolism." Oncotarget **7**(32): 52364-52374.
- Kikuchi, S., K. Honda, Y. Handa, H. Kato, K. Yamashita, T. Umaki, M. Shitashige, M. Ono, A. Tsuchida, T. Aoki, S. Hirohashi and T. Yamada (2007). "Serum albumin-associated peptides of patients with uterine endometrial cancer." Cancer Sci **98**(6): 822-829.
- Kliemann, N., V. Viallon, N. Murphy, R. J. Beeken, J. A. Rothwell, S. Rinaldi, N. Assi, E. H. van Roekel, J. A. Schmidt, K. B. Borch, C. Agnoli, A. H. Rosendahl, H. Sartor, J. M. Huerta, A. Tjønneland, J. Halkjær, B. Bueno-de-Mesquita, A. Gicquiau, D. Achaintre, K. Aleksandrova, M. B. Schulze, A. K. Heath, K. K. Tsilidis, G. Masala, S. Panico, R. Kaaks, R. T. Fortner, B. Van Guelpen, L. Dossus, A. Scalbert, H. C. Keun, R. C. Travis, M. Jenab, M. Johansson, P. Ferrari and M. J. Gunter (2021). "Metabolic signatures of greater body size and their associations with risk of colorectal and endometrial cancers in the European Prospective Investigation into Cancer and Nutrition." BMC Med **19**(1): 101.
- Knific, T., K. Vouk, Š. Smrkolj, C. Prehn, J. Adamski and T. L. Rižner (2018). "Models including plasma levels of sphingomyelins and phosphatidylcholines as diagnostic and prognostic biomarkers of endometrial cancer." J Steroid Biochem Mol Biol **178**: 312-321.
- Kozar, N., K. Kruusmaa, A. Dovnik, M. Bitenc, R. Argamasilla, A. Adsuar, N. Goswami, I. Takač and D. Arko (2021). "Identification of novel diagnostic biomarkers in endometrial cancer using targeted metabolomic profiling." Adv Med Sci **66**(1): 46-51.
- Kurimchak, A. M., V. Kumar, C. Herrera-Montavez, K. J. Johnson, N. Srivastava, K. Davarajan, S. Peri, K. Q. Cai, G. M. Mantia-Smaldone and J. S. Duncan (2020). "Kinome Profiling of Primary Endometrial Tumors Using Multiplexed Inhibitor Beads and Mass Spectrometry Identifies SRPK1 as Candidate Therapeutic Target." Molecular and Cellular Proteomics **19**(12): 2068-2089.
- Kurimchak, A. M., V. Kumar, C. Herrera-Montavez, K. J. Johnson, N. Srivastava, K. Davarajan, S. Peri, K. Q. Cai, G. M. Mantia-Smaldone and J. S. Duncan (2068). "Kinome Profiling of Primary Endometrial Tumors Using Multiplexed Inhibitor Beads and Mass Spectrometry Identifies SRPK1 as Candidate Therapeutic Target." Molecular and Cellular Proteomics **19**(12): 2068-2089.
- Li, Z., C. Huang, S. Bai, X. Pan, R. Zhou, Y. Wei and X. Zhao (2008). "Prognostic evaluation of epidermal fatty acid-binding protein and calcyphosine, two proteins implicated in endometrial cancer using a proteomic approach." Int J Cancer **123**(10): 2377-2383.
- Li, Z., W. Min, C. Huang, S. Bai, M. Tang and X. Zhao (2010). "Proteomics-based approach identified differentially expressed proteins with potential roles in endometrial carcinoma." Int J Gynecol Cancer **20**(1): 9-15.
- Li, Z., X. Zhao, S. Bai, Z. Wang, L. Chen, Y. Wei and C. Huang (2008). "Proteomics identification of cyclophilin a as a potential prognostic factor and therapeutic target in endometrial carcinoma." Mol Cell Proteomics **7**(10): 1810-1823.
- Lintel, N. J., S. A. Luebker, S. M. Lele and S. A. Koepsell (2018). "MVP immunohistochemistry is a useful adjunct in distinguishing leiomyosarcoma from leiomyoma and leiomyoma with bizarre nuclei." Hum Pathol **73**: 122-127.
- Liu, Z., Z. Hong and P. Qu (2020). "Proteomic Analysis of Human Endometrial Tissues Reveals the Roles of PI3K/AKT/mTOR Pathway and Tumor Angiogenesis Molecules in the Pathogenesis of Endometrial Cancer." BioMed Research International(pagination).
- Lunde, S., H. T. Nguyen, K. K. Petersen, L. Arendt-Nielsen, H. B. Krarup and E. Søgaard-Andersen (2020). "Chronic Postoperative Pain After Hysterectomy for Endometrial Cancer: A Metabolic Profiling Study." Mol Pain **16**: 1744806920923885.
- Martinez-Garcia, E., A. Lesur, L. Devis, S. Cabrera, X. Matias-Guiu, M. Hirschfeld, J. Asberger, J. van Oostrum, M. L. A. Casares de Cal, A. Gomez-Tato, J. Reventos, B. Domon, E. Colas and A. Gil-Moreno (2017). "Targeted Proteomics Identifies Proteomic Signatures in Liquid Biopsies of the Endometrium to Diagnose Endometrial Cancer and Assist in the Prediction of the Optimal Surgical Treatment." Clin Cancer Res **23**(21): 6458-6467.
- Martinez-Garcia, E., A. Lesur, L. Devis, A. Campos, S. Cabrera, J. van Oostrum, X. Matias-Guiu, A. Gil-Moreno, J. Reventos, E. Colas and B. Domon (2016). "Development of a sequential workflow based on LC-PRM for the verification of endometrial cancer protein biomarkers in uterine aspirate samples." Oncotarget **7**(33): 53102-53115.
- Mauland, K. K., Z. Ju, I. L. Tangen, A. Berg, K. H. Kalland, A. M. Oyan, L. Bjorge, S. N. Westin, C. Krakstad, J. Trovik, G. B. Mills, E. A. Hoivik and H. M. J. Werner (2017). "Proteomic profiling of endometrioid endometrial cancer reveals differential expression of hormone receptors and MAPK signaling proteins in obese versus non-obese patients." Oncotarget **8**(63): 106989-107001.
- Mittal, P., M. Klingler-Hoffmann, G. Arentz, L. Winderbaum, G. Kaur, L. Anderson, J. Scurry, Y. Leung, C. J. Stewart, J. Carter, P. Hoffmann and M. K. Oehler (2017). "Annexin A2 and alpha actinin 4 expression correlates with metastatic potential of primary endometrial cancer." Biochim Biophys Acta Proteins Proteom **1865**(7): 846-857.
- Mittal, P., M. Klingler-Hoffmann, G. Arentz, L. Winderbaum, N. A. Lokman, C. Zhang, L. Anderson, J. Scurry, Y. Leung, C. J. Stewart, J. Carter, G. Kaur, M. K. Oehler and P. Hoffmann (2016). "Lymph node metastasis of primary endometrial cancers: Associated proteins revealed by MALDI imaging." Proteomics **16**(11-12): 1793-1801.
- Mu, A. K., B. K. Lim, N. Aminudin, O. H. Hashim and A. S. Shuib (2016). "Application of SELDI-TOF in N-glycopeptides profiling of the urine from patients with endometrial, ovarian and cervical cancer." Arch Physiol Biochem **122**(3): 111-116.
- Mu, A. K., B. K. Lim, O. H. Hashim and A. S. Shuib (2012). "Detection of differential levels of proteins in the urine of patients with endometrial cancer: analysis using two-dimensional gel electrophoresis and o-glycan binding lectin." Int J Mol Sci **13**(8): 9489-9501.
- Njoku, K., A. E. Campbell, B. Geary, M. L. MacKintosh, A. E. Derbyshire, S. J. Kitson, V. N. Sivalingam, A. Pierce, A. D. Whetton and E. J. Crosbie (2021). "Metabolomic Biomarkers for the Detection of Obesity-Driven Endometrial Cancer." Cancers (Basel) **13**(4).
- Qiu, F., Y. H. Gao, C. G. Jiang, Y. P. Tian and X. J. Zhang (2010). "Serum proteomic profile analysis for endometrial carcinoma detection with MALDI-TOF MS." Arch Med Sci **6**(2): 245-252.
- Schuhn, A., T. W. Tobar, A. W. Gahlawat, J. Hauke, L. Baumann, J. G. Okun and J. Nees (2022). "Potential of blood-based biomarker approaches in endometrium and breast cancer: a case-control comparison study." Arch Gynecol Obstet.
- Shafiee, M. N., C. A. Ortori, D. A. Barrett, N. P. Mongan, J. Abu and W. Atiomo (2020). "Lipidomic Biomarkers in Polycystic Ovary Syndrome and Endometrial Cancer." Int J Mol Sci **21**(13).
- Shan, N., W. Zhou, S. Zhang and Y. Zhang (2016). "Identification of HSPA8 as a candidate biomarker for endometrial carcinoma by using iTRAQ-based proteomic analysis." Onco Targets Ther **9**: 2169-2179.
- Shao, X., K. Wang, X. Liu, C. Gu, P. Zhang, J. Xie, W. Liu, L. Sun, T. Chen and Y. Li (2016). "Screening and verifying endometrial carcinoma diagnostic biomarkers based on a urine metabolomic profiling study using UPLC-Q-TOF/MS." Clin Chim Acta **463**: 200-206.
- Shi, K., Q. Wang, Y. Su, X. Xuan, Y. Liu, W. Chen, Y. Qian and G. E. Lash (2018). "Identification and functional analyses of differentially expressed metabolites in early stage endometrial carcinoma." Cancer Sci **109**(4): 1032-1043.
- Skorupa, A., M. Ponski, M. Ciszek, B. Cichon, M. Klimek, A. Witek, S. Pakulo, L. Boguszewicz and M. Sokol (2021). "Grading of endometrial cancer using <sup>1</sup>H HR-MAS NMR-based metabolomics." Scientific reports **11(1)**: 18160.
- Strand, E., I. L. Tangen, K. E. Fasmer, H. Jacob, M. K. Halle, E. A. Hoivik, B. Delvoux, J. Trovik, I. S. Haldorsen, A. Romano and C. Krakstad (2019). "Blood Metabolites Associate with Prognosis in Endometrial Cancer." Metabolites **9**(12).
- Tarney, C. M., G. Wang, N. W. Bateman, K. A. Conrads, M. Zhou, B. L. Hood, J. Loffredo, C. Tian, K. M. Darcy, C. A. Hamilton, Y. Casablanca, A. Lokshin, T. P. Conrads and G. L. Maxwell (2019). "Biomarker panel for early detection of endometrial cancer in the Prostate, Lung, Colorectal, and Ovarian cancer screening trial." Am J Obstet Gynecol **221**(5): 472.e471-472.e410.
- Troisi, J., A. Raffone, A. Travaglino, G. Belli, C. Belli, S. Anand, L. Giugliano, P. Cavallo, G. Scala, S. Symes, S. Richards, D. Adair, A. Fasano, V. Bottigliero and M. Guida (2020). "Development and Validation of a Serum Metabolomic Signature for Endometrial Cancer Screening in Postmenopausal Women." JAMA Netw Open **3**(9): e2018327.
- Troisi, J., L. Sarno, A. Landolfi, G. Scala, P. Martinelli, R. Venturella, A. Di Cello, F. Zullo and M. Guida (2018). "Metabolomic Signature of Endometrial Cancer." J Proteome Res **17**(2): 804-812.
- Trousil, S., P. Lee, D. J. Pinato, J. K. Ellis, R. Dina, E. O. Aboagye, H. C. Keun and R. Sharma (2014). "Alterations of choline phospholipid metabolism in endometrial cancer are caused by choline kinase alpha overexpression and a hyperactivated deacylation pathway." Cancer Res **74**(23): 6867-6877.
- Ura, B., S. Biffi, L. Monasta, G. Arrigoni, I. Battisti, G. Di Lorenzo, F. Romano, M. Aloisio, F. Celsi, R. Addobbati, F. Valle, E. Rampazzo, M. Brucale, A. Ridolfi, D. Licastro and G. Ricci (2021). "Two dimensional-difference in gel electrophoresis (2d-dige) proteomic approach for the identification of biomarkers in endometrial cancer serum." Cancers **13**(14).
- Ura, B., V. Capaci, M. Aloisio, G. Di Lorenzo, F. Romano, G. Ricci and L. Monasta (2022). "A Targeted Proteomics Approach for Screening Serum Biomarkers Observed in the Early Stage of Type I Endometrial Cancer." Biomedicines **10**(8).
- Ura, B., L. Monasta, G. Arrigoni, C. Franchin, O. Radillo, I. Peterlunger, G. Ricci and F. Scrimin (2017). "A proteomic approach for the identification of biomarkers in endometrial cancer uterine aspirate." Oncotarget **8**(65): 109536-109545.
- Uyar, D. S., Y. W. Huang, M. A. Chesnik, N. B. Doan and S. P. Mirza (2021). "Comprehensive serum proteomic analysis in early endometrial cancer." Journal of Proteomics **234**(no pagination).
- Voisin, S. N., O. Krakovska, A. Matta, L. V. DeSouza, A. D. Romaschin, T. J. Colgan and K. W. Siu (2011). "Identification of novel molecular targets for endometrial cancer using a drill-down LC-MS/MS approach with iTRAQ." PLoS One **6**(1): e16352.
- Wang, Y. S., R. Cao, H. Jin, Y. P. Huang, X. Y. Zhang, Q. Cong, Y. F. He and C. J. Xu (2011). "Altered protein expression in serum from endometrial hyperplasia and carcinoma patients." J Hematol Oncol **4**: 15.
- Yan, X., W. Zhao, J. Wei, Y. Yao, G. Sun, L. Wang, W. Zhang, S. Chen, W. Zhou, H. Zhao, X. Li, Y. Xiao and Y. Li (2022). "A serum lipidomics study for the identification of specific biomarkers for endometrial polyps to distinguish them from endometrial cancer or hyperplasia." Int J Cancer **150**(9): 1549-1559.
- Yi, R., L. Xie, X. Wang, C. Shen, X. Chen and L. Qiao (2022). "Multi-Omic Profiling of Multi-Biosamples Reveals the Role of Amino Acid and Nucleotide Metabolism in Endometrial Cancer." Front Oncol **12**: 861142.
- Yi, R., L. Xie, X. Wang, C. Shen, X. Chen and L. Qiao (2022). "Multi-Omic Profiling of Multi-Biosamples Reveals the Role of Amino Acid and Nucleotide Metabolism in Endometrial Cancer." Frontiers in Oncology **12**(no pagination).
- Yoshizaki, T., T. Enomoto, R. Nakashima, Y. Ueda, H. Kanao, K. Yoshino, M. Fukumoto, Y. Yoneda, G. S. Buzard and Y. Murata (2005). "Altered protein expression in endometrial carcinogenesis." Cancer Lett **226**(2): 101-106.
- Zhu, L. R., W. Y. Zhang, L. Yu, Y. H. Zheng, J. Hu and Q. P. Liao (2008). "Proteiomic patterns for endometrial cancer using SELDI-TOF-MS." J Zhejiang Univ Sci B **9**(4): 286-290.
- Zhu, L. R., W. Y. Zhang, L. Yu, Y. H. Zheng, J. Z. Zhang and Q. P. Liao (2006). "Serum proteomic features for detection of endometrial cancer." Int J Gynecol Cancer **16**(3): 1374-1378.
